# DUSP8 induces TGF-**β**–stimulated *IL-9* transcription and Th9-mediated allergic inflammation by promoting nuclear export of Pur-**α**

**DOI:** 10.1172/JCI166269

**Published:** 2023-11-01

**Authors:** Huai-Chia Chuang, Chia-Hsin Hsueh, Pu-Ming Hsu, Ching-Yi Tsai, Ying-Chun Shih, Hsien-Yi Chiu, Yi-Ming Chen, Wen-Kuang Yu, Ming-Han Chen, Tse-Hua Tan

**Affiliations:** 1 Immunology Research Center, National Health Research Institutes, Zhunan, Taiwan.; 2 Department of Dermatology, National Taiwan University Hospital Hsin-Chu Branch, Hsinchu, Taiwan.; 3 Division of Allergy, Immunology, and Rheumatology, Taichung Veterans General Hospital, Taichung, Taiwan.; 4 Department of Chest Medicine and; 5 Division of Allergy, Immunology, and Rheumatology, Taipei Veterans General Hospital, Taipei, Taiwan.

**Keywords:** Inflammation, Phosphoprotein phosphatases, Signal transduction, T cells

## Abstract

Dual-specificity phosphatase 8 (DUSP8) is a MAPK phosphatase that dephosphorylates and inactivates the kinase JNK. DUSP8 is highly expressed in T cells; however, the in vivo role of DUSP8 in T cells remains unclear. Using T cell–specific *Dusp8* conditional KO (T-*Dusp8* cKO) mice, mass spectrometry analysis, ChIP-Seq, and immune analysis, we found that DUSP8 interacted with Pur-α, stimulated interleukin-9 (*IL-9*) gene expression, and promoted Th9 differentiation. Mechanistically, DUSP8 dephosphorylated the transcriptional repressor Pur-α upon TGF-β signaling, leading to the nuclear export of Pur-α and subsequent *IL-9* transcriptional activation. Furthermore, *Il-9* mRNA levels were induced in *Pur-*α–deficient T cells. In addition, T-*Dusp8*–cKO mice displayed reduction of IL-9 and Th9-mediated immune responses in the allergic asthma model. Reduction of *Il-9* mRNA levels in T cells and allergic responses of T-Dusp8–cKO mice was reversed by *Pur-*α knockout. Remarkably, DUSP8 protein levels and the DUSP8–Pur-α interaction were indeed increased in the cytoplasm of T cells from people with asthma and patients with atopic dermatitis. Collectively, DUSP8 induces TGF-β–stimulated *IL-9* transcription and Th9-induced allergic responses by inhibiting the nuclear translocation of the transcriptional repressor Pur-α. DUSP8 may be a T-cell biomarker and therapeutic target for asthma and atopic dermatitis.

## Graphical abstract

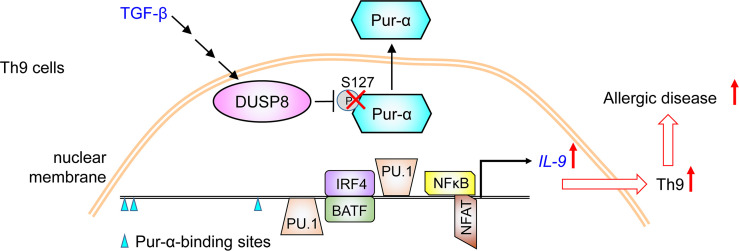

## Introduction

Dual-specificity phosphatase 8, or DUSP8 — also named M3/6 and hVH5 — is a member of the dual-specificity phosphatase (DUSP) family phosphatases (1–3). The subcellular localization of DUSP8 is mainly in the cytoplasm and nucleus (4). DUSP8 plays an important role in cardiac ventricular remodeling (5). DUSP8 dephosphorylates and inactivates the kinase JNK (JNK1 and JNK2 isoforms), specifically, but not p38 and ERK, in Jurkat T cells (6) and 3T3-L1 adipocytes (7). *Dusp8*-deficient mice show hyperactivation of JNK signaling in the hypothalamus and display glucose intolerance (8). TNF-α induces the expression of DUSP8, which dephosphorylates JNK and subsequently inhibits the production of proinflammatory cytokines MCP-1 and IL-6 in 3T3-L1 adipocytes (7). To date, the in vivo role of DUSP8 in T cell functions and immune responses has not been investigated.

IL-9–producing CD4^+^ T (Th9) cells induce allergies, such as asthma and atopic dermatitis, and inflammation/autoimmune diseases, such as inflammatory bowel diseases, multiple sclerosis, systemic lupus erythematosus, and psoriasis (9–11). IL-9 induction promotes recruitment of mast cells/eosinophils and induces activation of interstitial macrophages in the inflamed tissues, resulting in the expansion of allergic inflammation (12, 13). Besides its proinflammation function, IL-9 also plays dual roles in antitumor immunity (9, 14) and protumor immunity (15). Both IL-4–induced IRF-4, as well as TGF-β–induced Pu.1 are required for Th9 cell differentiation (10). Additional transcription factors such as STAT6, GATA3, BATF, NF-AT, and NF-κB also regulate *IL-9* transcription (9, 16). In addition to IL-4-STAT6–induced IRF-4 activation, IL-4–STAT6 signaling also downregulates the Foxp3-inhibited Th9 differentiation (17). The DNA-binding protein Pur-α acts as a transcriptional repressor (18) or a activator (19) for its target genes. To date, the role of Pur-α in controlling *IL-9* transcription is unknown. Here, we report that DUSP8 dephosphorylates the transcriptional repressor Pur-α in T cells, contributing to the induction of *IL-9* transcription, Th9 cell differentiation, and Th9-mediated allergic responses.

## Results

### T cell development is normal in T cell–specific Dusp8-cKO mice.

To study the in vivo role of DUSP8 in T cell functions, we generated T cell–specific *Dusp8* conditional KO (T-*Dusp8* cKO [*Dusp8^fl/fl^;Cd4-Cre*]) mice by crossing floxed *Dusp8* mice and *Cd4-Cre* transgenic mice (Supplemental Figure 1, A and B; supplemental material available online with this article; https://doi.org/10.1172/JCI166269DS1). DUSP8 ablation in peripheral blood T cells of T-*Dusp8*–cKO mice was confirmed by real-time PCR and immunoblotting analysis (Supplemental Figure 1, C and D). Four-week-old T-*Dusp8*–cKO mice displayed normal T cell development in the thymus, as well as normal peripherally derived natural Treg (nTreg) populations in the spleen and lymph nodes (Supplemental Figure 2, A–F).

### T cell profiles and T cell receptor–induced cytokines of T-Dusp8–cKO mice are similar to those of WT mice.

**Figure 1:**
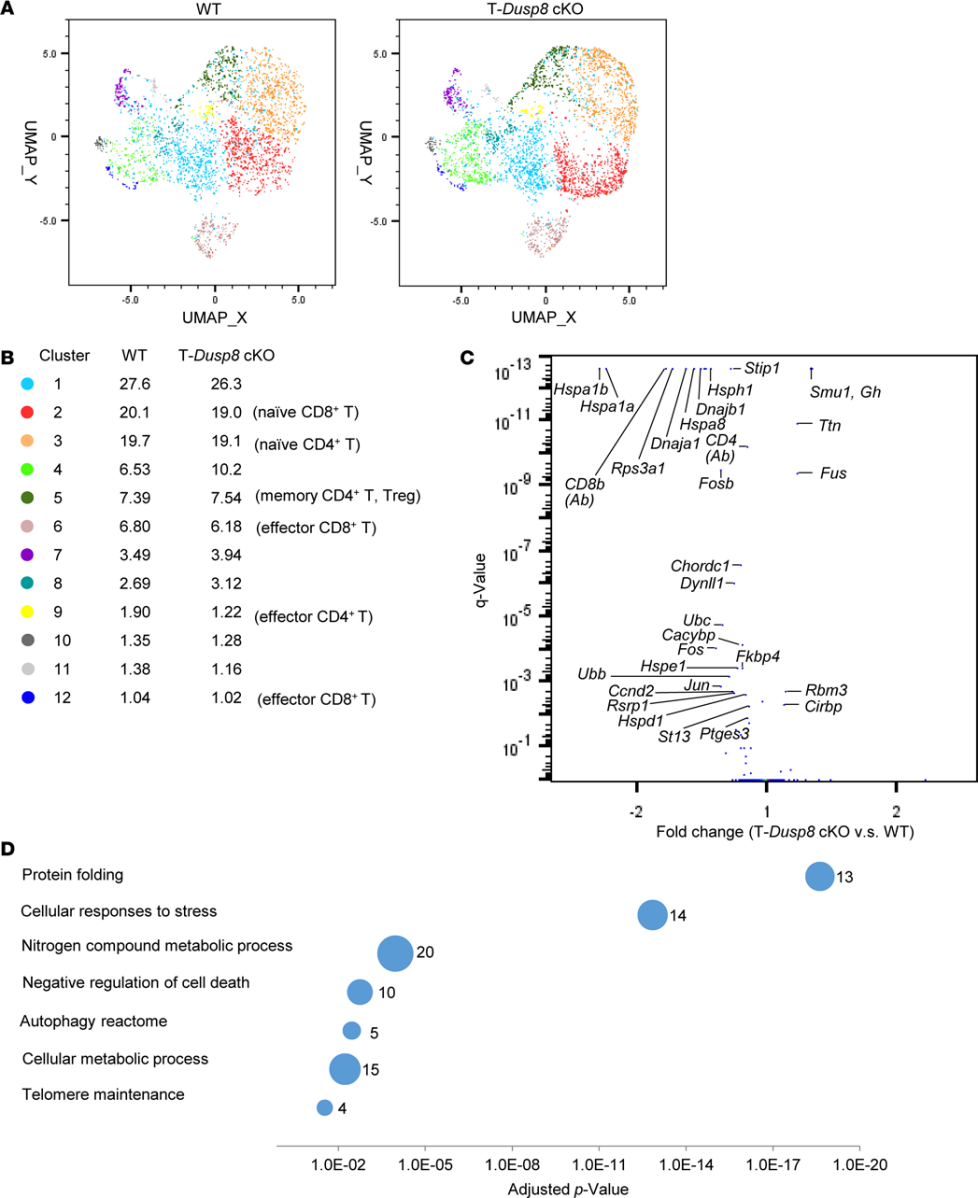
Basal T cell profiles of T-*Dusp8*–cKO mice are similar to those of WT mice. (**A** and **B**) Uniform Manifold Approximation and Projection (UMAP) plot of the scRNA-Seq data showed the distribution and classification of unstimulated T cells from T-*Dusp8*–cKO and WT mice. (**C**) The volcano plot of the selected differentially expressed genes in the unstimulated T cells of T-*Dusp8*–cKO and WT mice. Q value was determined using Fisher’s exact test. (**D**) KEGG pathway enrichment analysis of 28 downregulated genes in unstimulated T cells of T-*Dusp8*–cKO mice compared with WT mice. The different classification pathways are listed on the left of the plot. Varied numbers of genes enriched in individual pathways are presented by different diameter sizes and numbers for individual dots. T-*Dusp8* cKO, T cell–specific *Dusp8* cKO (*Dusp8^fl/fl^*;*Cd4*-*Cre*); WT (*Dusp8^fl/fl^*).

To investigate T cell profiles and potential immune phenotypes of T-*Dusp8*–cKO mice, unstimulated T cells from the spleen and lymph nodes of 6-month-old mice were subjected to single cell–RNA-Seq (scRNA-seq) analysis. Dimensionality reduction and clustering analyses showed that frequencies of individual T cell clusters of mice are comparable between WT and T-*Dusp8*–cKO mice (Figure 1, A and B). Notably, expression levels of 28 genes were decreased, while 6 other genes were increased, in T cells of T-*Dusp8*–cKO mice (Figure 1C). However, the fold changes on the mRNA levels of these 34 dysregulated genes were very modest (Figure 1C). Protein levels of CD4 and CD8 detected by the AbSeq antibody-oligonucleotide conjugates were decreased in T-*Dusp8*–cKO mice (Figure 1C); nevertheless, the frequencies of CD4^+^ T cells and CD8^+^ T cells were unchanged in T-*Dusp8*–cKO mice (Supplemental Figure 2B). Moreover, Kyoto Encyclopedia of Genes and Genomes (KEGG) pathway analyses revealed that the 28 downregulated genes in T-*Dusp8*–cKO T cells belong to protein folding signaling, stress-stimulated cellular responses, cell death/autophagy pathways, and metabolic pathways (Figure 1D). Among these DUSP8-regulated pathways, none of the pathways are associated with T cell–mediated immune responses. These results suggest that basal T cell subsets and immune phenotypes in the absence of stimulation are not affected by DUSP8 ablation.

To study whether T cell receptor (TCR) signaling–stimulated cytokine production is regulated by DUSP8, supernatants from TCR-stimulated splenic T cells of WT and T-*Dusp8*–cKO mice were subjected to cytokine array analysis. Among the 111 cytokines examined, several cytokines (including IL-2, IFN-γ, IL-10, and GM-CSF) were increased by TCR stimulation; however, none of these cytokines were markedly affected by *Dusp8* cKO in T cells (Supplemental Figure 3A). ELISA data also confirmed that IL-2, IFN-γ, IL-4, and IL-17A levels from TCR-stimulated splenic T cells of T-*Dusp8*–cKO mice were not changed compared with those of WT T cells (Supplemental Figure 3B). Besides the 111 aforementioned cytokines, the protein levels of IL-9 from TCR-stimulated splenic T cells were undetectable, suggesting that TCR signaling is not sufficient to induce IL-9 production from splenic T cells. Moreover, the data suggest that DUSP8 in CD4^+^ T cells does not regulate the production of the T-cell cytokine IL-2, the Th1-cytokine IFN-γ, the Th2-cytokine IL-4, or the Th17-cytokine IL-17A. To further study whether DUSP8 regulates T cell–mediated immune responses, T-*Dusp8*–cKO mice were immunized with keyhole limpet hemocyanin (KLH), a T cell–dependent antigen, using alum as an adjuvant. Serum levels of individual KLH-specific antibodies in the mice after primary and secondary immunization were not markedly changed in T-*Dusp8*–cKO mice (Supplemental Figure 3C). Only the production of KLH-specific IgG1 antibody (Th2 marker) was slightly reduced (*P* = 0.216) by *Dusp8* cKO (Supplemental Figure 3C); however, the reduction was not statistically significant. These results suggest that DUSP8 does not play an important role in the regulation of TCR signaling.

### Th9 cell differentiation is attenuated in T cells from T-Dusp8–cKO mice.

**Figure 2:**
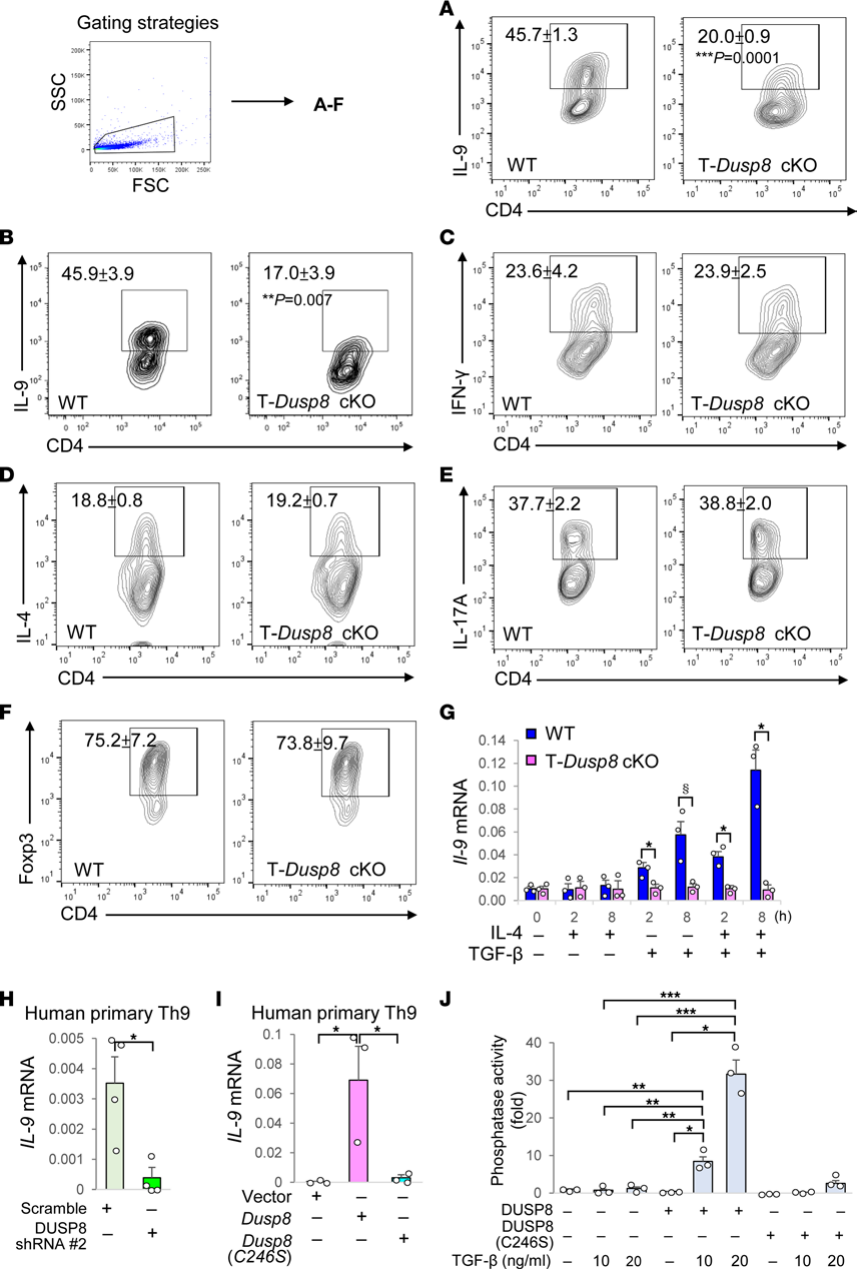
In vitro Th9 differentiation is reduced by *Dusp8* cKO. (**A**–**G**) Flow cytometry analyses of Th9 (CD4^+^IL-9^+^) (in vitro differentiated from splenic CD4^+^ T cells, (**A**), Th9 (CD4^+^IL-9^+^) (in vitro differentiated from splenic naive CD4^+^CD62L^+^ T cells, (**B**), Th1 (CD4^+^IFN-γ^+^) (**C**), Th2 (CD4^+^IL-4^+^) (**D**), Th17 (CD4^+^IL-17A^+^) (**E**), and Treg (CD4^+^Foxp3^+^) (**F**) cells of in vitro differentiated T cells. Mean ± SEM are shown. Data (**C**–**F**) were not significantly changed in T-*Dusp8*–cKO T cells. (**G**) Real-time PCR of *Il-9* mRNA levels in T-*Dusp8* cKO or WT T cells stimulated with IL-4 (20 ng/mL) and/or TGF-β (50 ng/mL) for 2 or 8 hours. The expression levels of *Il-9* were normalized to *Srp72* levels. Mean ± SEM are shown. *n* = 3. *P < 0.05 (2-tailed Student’s *t* test); ^§^*P* < 0.05 (1-tailed Student’s *t* test). (**H** and **I**) Real-time PCR of *IL-9* mRNA levels in DUSP8 shRNA knocked-down human primary Th9 cells (**H**) or DUSP8-overexpressing human primary Th9 cells (**I**). Human primary T cells were cultured under Th9 polarizing conditions for 5 days, followed by transfection with indicated plasmids. The expression levels of *IL-9* were normalized to *GAPDH* levels. Mean ± SEM are shown. *n* = 3. (**J**) In vitro phosphatase assays of purified DUSP8 proteins isolated from protein lysates of Myc-DUSP8 or Myc-DUSP8 (C246S) phosphatase-dead mutant-expressing J-TAg T cells stimulated with TGF-β (10 or 20 ng/mL). *n* = 3. Results (mean ± SEM) are presented relative to those of vector controls. T-*Dusp8* cKO, T cell-specific *Dusp8* cKO (*Dusp8^fl/fl^*;*Cd4-Cre*); WT (*Dusp8^fl/fl^*); DUSP8 (C246S), DUSP8 phosphatase-dead mutant. For (**H**–**J**), *P < 0.05; **P < 0.01; ***P < 0.001 (1-way ANOVA and Tukey’s posthoc test). Data shown are representative of 3 independent experiments.

To investigate whether DUSP8 plays a role in T-cell differentiation, we performed T helper (Th) cell differentiation in vitro. Remarkably, in vitro Th9 differentiation was reduced using splenic CD4^+^ T cells from T-*Dusp8*–cKO mice (Figure 2, A and B), whereas differentiation of Th1, Th2, Th17, or Treg was not affected (Figure 2, C–F). Because TGF-β or IL-4 treatment is critical for Th9 differentiation, we examined the IL-9 production in T cells of T-*Dusp8*–cKO mice under TGF-β or IL-4 stimulation. Strikingly, real-time PCR data showed that *Il-9* mRNA induction in murine primary T cells stimulated by TGF-β alone or IL-4 plus TGF-β treatment, but not IL-4 alone, was abolished by *Dusp8* cKO (Figure 2G). TGF-β–induced *Il-9* mRNA levels in human Jurkat T cells were decreased by DUSP8 shRNA knockdown (Supplemental Figure 4A); conversely, the levels were enhanced by DUSP8 overexpression (Supplemental Figure 4B). Consistently, *IL-9* mRNA levels were decreased by DUSP8 shRNA knockdown (Figure 2H) and induced by DUSP8 overexpression (Figure 2I) in human primary T cells under Th9 polarizing conditions (Supplemental Figure 4C). Moreover, DUSP8 phosphatase activity was induced by TGF-β signaling (Figure 2J). These results suggest that DUSP8 plays an important role in controlling *IL-9* gene expression in TGF-β-stimulated T cells.

### DUSP8 induces IL-9 production in T cells by inhibiting Pur-α function.

**Figure 3:**
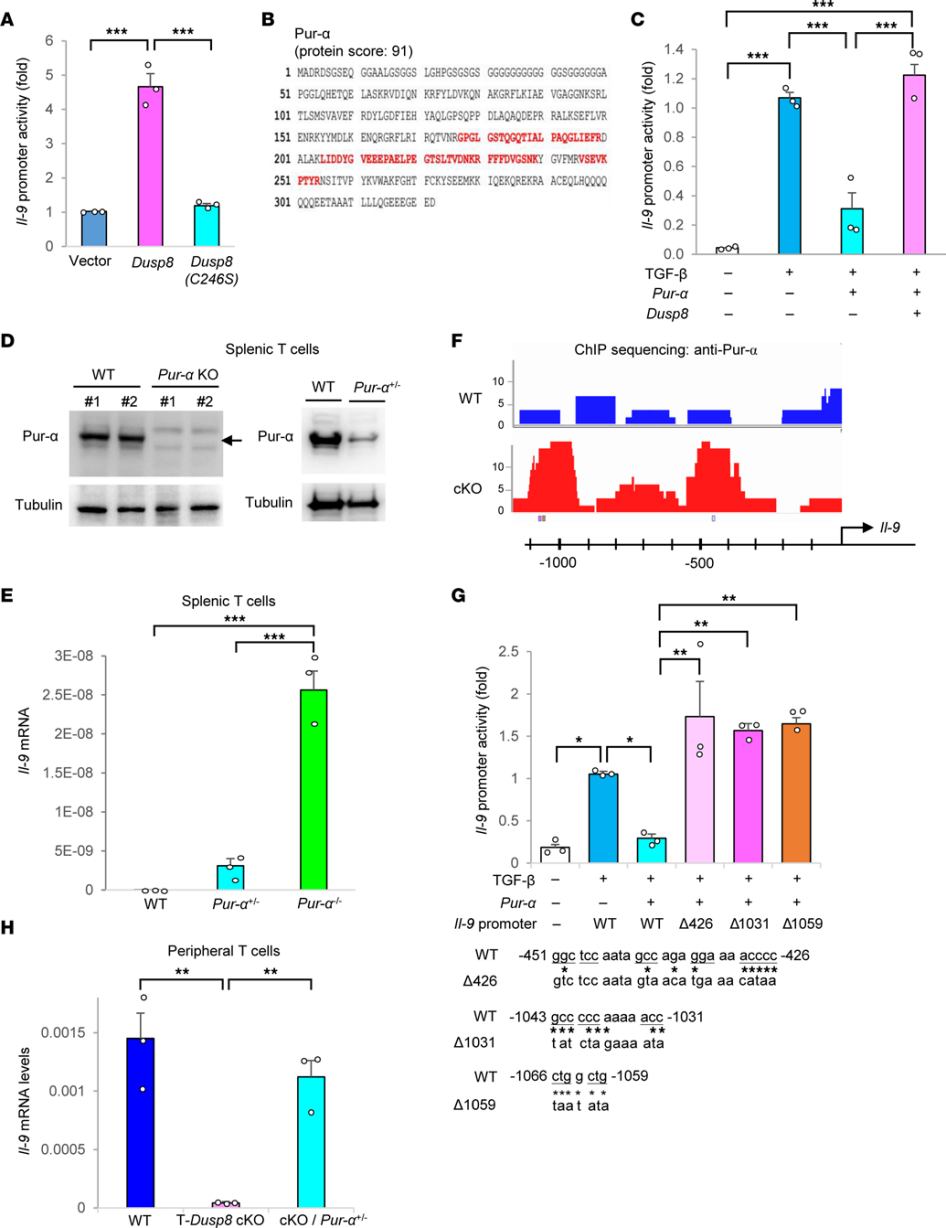
DUSP8 promotes *IL-9* transcription in T cells by blocking Pur-α suppressor function. (**A**) Luciferase reporter activity of the *Il-9* promoter. Jurkat T cells were cotransfected with the *Il-9* promoter (–1,097 to +26)-luciferase construct plus the plasmid encoding either DUSP8 WT or phosphatase-dead (C246S) mutant. T cells were stimulated with TGF-β. *n* = 3. (**B**) Identification of Pur-α as a DUSP8-interacting protein by mass spectrometry-based proteomics. Matched Pur-α peptides were shown in red. (**C**) Luciferase reporter activity of the *Il-9* promoter in TGF-β-stimulated Jurkat T cells cotransfected with Myc-*Dusp8* and Flag-*Pur-*α plasmids. *n* = 3. (**D**) Ablation or reduction of Pur-α proteins in splenic T cells of *Pur-*α homozygous KO (*Pur-*α^–/–^) mice or heterozygous KO (*Pur-*α^+/–^) mice were verified by immunoblot analysis. (**E**) Real-time PCR of basal *Il-9* mRNA levels in splenic T cells isolated from *Pur-*α heterozygous/homozygous KO and WT mice. *Il-9* mRNA levels were normalized to *Srp72* levels. *n* = 3. (**F**) The binding of Pur-α to the *Il-9* promoter in TGF-β-stimulated murine T cells was analyzed by ChIP-Seq using anti-Pur-α antibody. (**G**) Luciferase reporter activity of the *Il-9* promoter in TGF-β-stimulated Jurkat T cells cotransfected with *Pur-*α plasmid and the *Il-9* promoter-driven luciferase reporter plasmid containing WT or individually mutated Pur-α-binding repeats. *n* = 3. WT and mutated sequences (Δ426, Δ1031, Δ1059) of the 3 putative Pur-α-binding elements (underlined) are shown at the bottom. (**H**) Real-time PCR of *Il-9* mRNA levels in WT, T-*Dusp8* cKO, or *Dusp8^fl/fl^*;*Cd4-Cre*;*Pur-*α^+/–^ peripheral blood T cells stimulated with TGF-β (50 ng/mL) and IL-4 (20 ng/mL) for 8 hours. *n* = 3. Mean ± SEM are shown. *P < 0.05; **P < 0.01; ***P < 0.001 (1-way ANOVA and Tukey’s posthoc test). Data shown are representative of 3 independent experiments.

Next, we tested whether DUSP8 regulates IL-9 production through transcriptional activation of the *IL-9* promoter. *IL-9* promoter activity in Jurkat T cells was enhanced by DUSP8 overexpression but not by the DUSP8 (C246S) phosphatase–dead mutant (Figure 3A). To investigate the mechanism of DUSP8-induced *IL-9* transcription in T cells, we characterized DUSP8-interacting proteins by performing mass spectrometry–based proteomics using anti-myc (DUPS8)–immunocomplexes. Among DUSP8-interacting proteins, we identified a DNA-binding protein, Pur-α (Figure 3B), which acts as either a transcriptional repressor (18) or activator (19) for different target genes. Pur-α interacts with the coactivator Smad in myofibroblasts (18). TGF-β-stimulated Smad2 and Smad4 are required for *IL-9* transcription (20); however, it is unknown whether Pur-α regulates *IL-9* transcription. Next, we examined whether Pur-α regulates *IL-9* transcription using luciferase reporter assays of the *IL-9* promoter. TGF-β-stimulated *IL-9* promoter activity was inhibited by Pur-α overexpression in Jurkat T cells (Figure 3C). Notably, Pur-α–inhibited *IL-9* transcription was reversed by DUSP8 overexpression (Figure 3C). To further confirm the suppressive function of Pur-α in the regulation of IL-9 transcription, we generated *Pur-*α KO mice and characterized *Pur-*α–KO T cells (Supplemental Figure 5, A and B). *Pur-*α homozygous–KO mice died prematurely at or before the age of 3 weeks. Due to the lack of mature *Pur-*α–KO mice, it was not feasible to perform in vitro Th9 differentiation assays or in vivo immunization studies on these mice. Nevertheless, we managed to perform immunoblotting and real-time PCR analyses using the scarce splenocytes from immature *Pur-*α–KO mice. Prior to premature death, the few available 3-week-old *Pur-*α homozygous–KO mice displayed normal T cell development in the thymus (Supplemental Figure 5, C and D). Splenic T cells of 3-week-old *Pur-*α homozygous–KO mice showed the anticipated deletion of *Pur-*α (Figure 3D) and a drastic induction of *Il-9* mRNA levels without any stimulation (Figure 3E). Notably, splenic T cells of *Pur-*α heterozygous–KO mice also showed an efficient reduction of Pur-α protein levels (Figure 3D) and enhancement of *Il-9* mRNA levels (Figure 3E). These results support that Pur-α acts as a repressor for *Il-9* transcription. Collectively, the above results suggest that Pur-α constitutively binds to the *Il-9* promoter and inhibits *Il-9* promoter activity in the absence of DUSP8.

To characterize the binding regions of Pur-α within the IL-9 gene locus, we performed ChIP-Seq using Pur-α immunocomplexes isolated from the lysates of TGF-β-stimulated T cells. The data showed the enhanced bindings of Pur-α to multiple genes in T cells of T-*Dusp8*–cKO mice, suggesting that Pur-α may be involved in multiple T cell signaling pathways (Supplemental Figure 6). Moreover, ChIP data showed enhanced Pur-α bindings to 2 distinct regions (-555 to -360 and -1,186 to -856) of the *Il-9* promoter in T-*Dusp8*–cKO mice (Figure 3F and Supplemental Figure 7A). After close examination of the *Il-9* promoter sequence for Pur-α–binding repeats, (GGN)n (19, 21, 22) or (CAG)n (23), we found 3 putative Pur-α–binding repeats coinciding with the 2 regions that showed the most enhanced Pur-α bindings to the *Il-9* promoter in T-*Dusp8*–cKO T cells detected by ChIP assays (Figure 3F and Supplemental Figure 7B). Furthermore, the TGF-β–stimulated *Il-9* reporter activity was suppressed by Pur-α overexpression, whereas the Pur-α-mediated suppression was reversed by individual mutations of the 3 putative Pur-α–binding repeats (-451 to -426, -1,043 to -1,031, -1,066 to -1,059) (Figure 3G). To verify that DUSP8 induces *Il-9* transcription by blocking the suppressive function of Pur-α, T-*Dusp8*–cKO mice were crossed with *Pur-*α heterozygous–KO mice. Due to the premature death of *Pur-*α homozygous-KO mice, we thus characterized T cells isolated from T-*Dusp8* cKO/*Pur-*α heterozygous–KO mice. Remarkably, the reduction of *Il-9* mRNA levels in peripheral T cells of T-*Dusp8*–cKO mice was efficiently reversed by *Pur-*α heterozygous KO (Figure 3H). Collectively, these results suggest that DUSP8 induces IL-9 production in T cells by blocking the binding of Pur-α to the *Il-9* promoter.

### DUSP8 dephosphorylates Pur-α at Ser-127 and induces its nuclear export.

**Figure 4:**
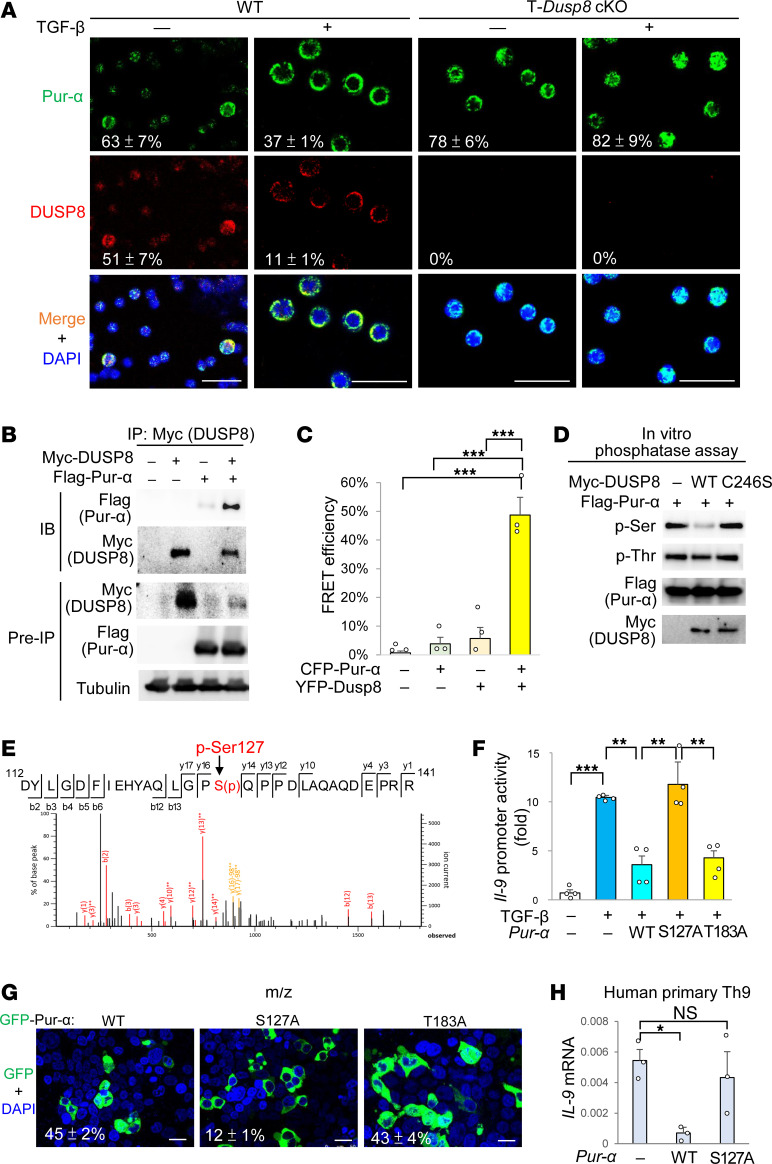
DUSP8 dephosphorylates Pur-α and induces its nuclear export. (**A**) Confocal microscopy analysis of endogenous Pur-α and DUSP8 in T cells from T-*Dusp8*–cKO or WT mice. T cell nucleus was stained with DAPI. Scale bars: 25 μm. Mean ± SEM of Pur-α nuclear localization (green over blue, %) or DUSP8 nuclear localization (red over blue, %) from 3 images are shown at the bottom. (**B**) Coimmunoprecipitations of Myc-tagged DUSP8 with Flag-tagged Pur-α proteins in the lysates of indicated HEK293T transfectants. (**C**) FRET analysis of HEK293T cells transfected with indicated plasmids encoding CFP-fused Pur-α or YFP-fused DUSP8 proteins. *n* = 3. (**D**) In vitro phosphatase assays of purified DUSP8 WT or phosphatase-dead (C246S) mutant, using purified Flag-tagged Pur-α proteins as substrates. Phosphorylation of Pur-α was determined by immunoblot analyses using anti-pan-phospho-serine (p-Ser) and anti-pan-phospho-threonine (p-Thr) antibodies. (**E**) Mass spectrometry analysis of the tryptic peptides of Pur-α to identify the peptide containing phosphorylated Ser127 residue. (**F**) Luciferase reporter activity of the *Il-9* promoter in TGF-β-stimulated Jurkat T cells cotransfected with *Il-9*-luciferase reporter plasmid and *Pur-*α WT or phospho-deficient mutant (S127A or T183A) plasmid. *n* = 4. (**G**) Confocal microscopy analysis of Pur-α WT or phospho-deficient mutant (S127A or T183A) in HEK293T cells. Cell nucleus was stained with DAPI. Scale bars: 25 μm. Mean ± SEM of Pur-α nuclear localization (green over blue, %) from 3 images are shown at the bottom. (**H**) Real-time PCR of *IL-9* mRNA levels in human primary Th9 cells transfected with *Pur-*α WT or phospho-deficient mutant (S127A) plasmid. *IL-9* mRNA levels were normalized to *GAPDH* levels. Means ± SEM are shown. *P* < 0.05; **P < 0.01; ***P < 0.001 (1-way ANOVA and Tukey’s posthoc test). Data shown (**A**–**D** and **F**–**H**) are representative of 3 independent experiments.

We next studied the mechanism by which DUSP8 regulates Pur-α suppressor to induce IL-9 transcription. Pur-α can translocate from the nucleus to the cytoplasm in neurons (24); therefore, we studied whether DUSP8 suppresses Pur-α binding to the *Il-9* promoter by regulating the subcellular localization of Pur-α. Confocal imaging showed that TGF-β treatment indeed induced Pur-α nuclear export in WT T cells; in contrast, Pur-α localized to the nucleus in T-*Dusp8*–cKO T cells (Figure 4A). Notably, the nuclear localizations of the Th9-related transcription factors PU.1, BATF, and IRF-4 in the in vitro differentiated Th9 cells were not significantly reduced by *DUSP8* cKO (Supplemental Figure 8, A and B). In addition, the mRNA levels of the Th9 suppressor *Foxp3* were not induced in the in vitro differentiated Th9 cells of T-*Dusp8*–cKO mice (Supplemental Figure 8C). These results suggest that DUSP8 may regulate Th9 differentiation mainly via inhibiting Pur-α. Our confocal imaging data support that DUSP8 interacts with Pur-α and induces Pur-α nuclear export. The interaction between DUSP8 and Pur-α was confirmed by co-IP analysis (Figure 4B) and fluorescence resonance energy transfer (FRET) analysis, which showed a direct interaction (< 10 nm) (Figure 4C). In situ proximity ligation assay (PLA) signals also showed a direct interaction (< 40 nm) between DUSP8 and Pur-α; these PLA signals were further enhanced by TGF-β stimulation in murine primary T cells (Supplemental Figure 9). Because DUSP8 phosphatase activity was required for *Il-9* transcriptional activation (Figure 3A), we studied whether DUSP8 directly dephosphorylates and subsequently induces Pur-α nuclear export. In vitro phosphatase assays using purified proteins showed that DUSP8 dephosphorylated Pur-α at a serine residue(s) but not threonine residue(s) (Figure 4D). To identify the DUSP8-targeted dephosphorylation residue on Pur-α, Pur-α proteins and in vitro DUSP8-dephosphorylated Pur-α proteins were isolated and subjected to mass spectrometry analyses. Ser127 on the Pur-α protein was identified as a DUSP8-dephosphorylated site (Figure 4E). Similar to DUSP8-dephosphorylated Pur-α protein, phospho-deficient Pur-α (S127A) mutant failed to inhibit *Il-9* promoter activity (Figure 4F) and localized to the cytoplasm (Figure 4G). Moreover, TGF-β-induced *IL-9* mRNA levels in human primary Th9 cells or human Jurkat T cells were inhibited by Pur-α overexpression but not by phospho-deficient Pur-α (S127A) mutation (Figure 4H and Supplemental Figure 10). Collectively, DUSP8 blocks Pur-α function by dephosphorylating Pur-α and promoting Pur-α nuclear export, thus derepressing *IL-9* transcription in T cells.

### DUSP8 plays a critical role in T cell–mediated allergic responses.

**Figure 5:**
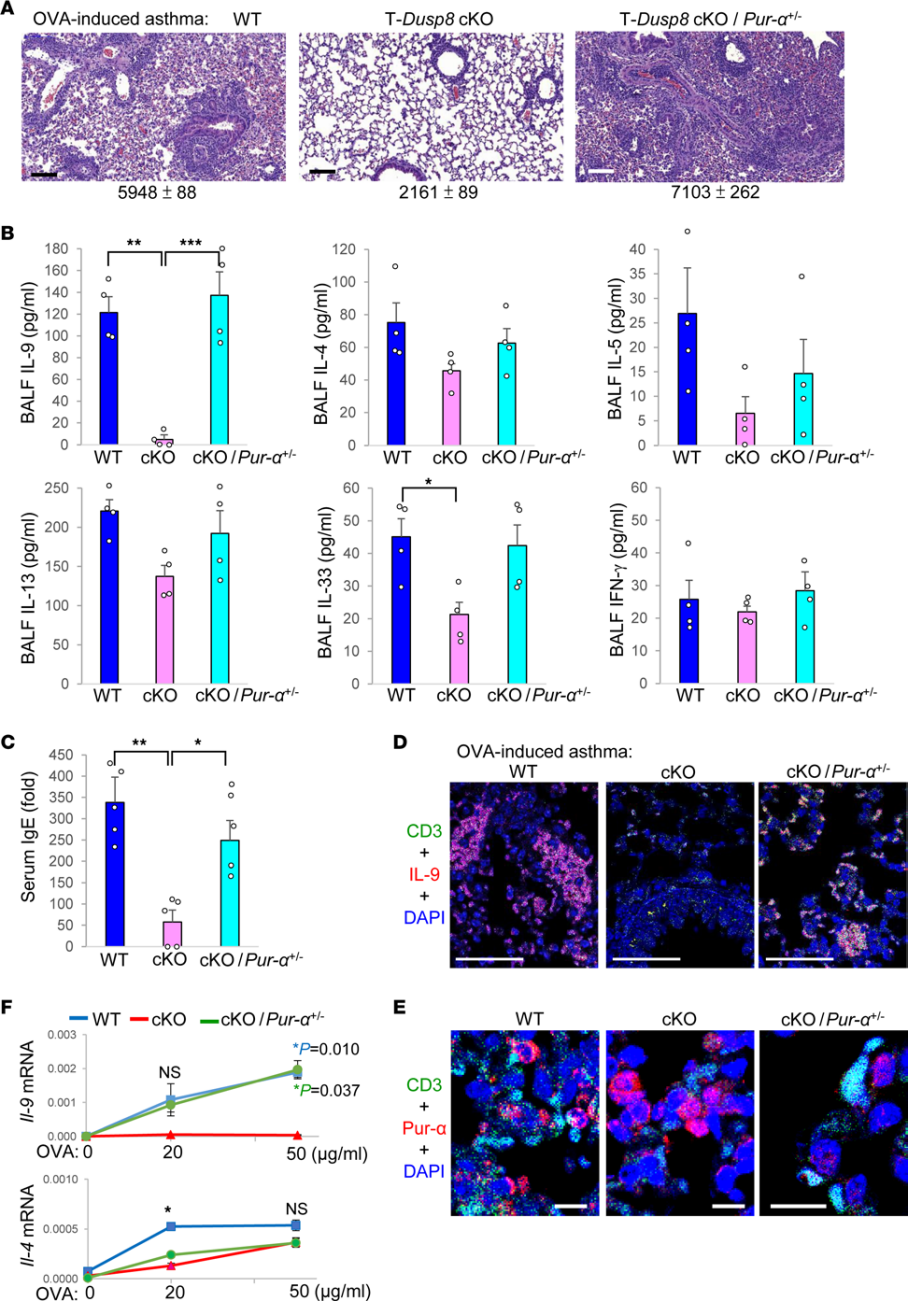
T cell–specific *Dusp8* cKO mice are resistant to OVA-induced allergic asthma. (**A**–**F**) Induction of OVA-induced asthma models in WT, T-*Dusp8*–cKO, or T-*Dusp8* cKO/*Pur-*α heterozygous–KO (cKO/*Pur-*α^+/–^) mice. H&E-stained sections of the lung tissues from mice on day 26 of the OVA-induced asthma model (**A**). Mean ± SEM of infiltrating immune cells from 3 images are shown below. Scale bars: 250 μm. The cytokine levels in the BALFs of mice on day 26 of the OVA-induced asthma model were determined using ELISA assays (**B**). *n* = 4. The OVA-specific serum IgE levels of mice on day 26 were determined by ELISA assays (**C**). Data are presented relative to the basal levels from a WT mouse. *n* = 5. Confocal microscopy analyses of CD3 (green), IL-9 (red), and DAPI in the lung tissues from mice on day 26 of the OVA-induced asthma model (**D**). Scale bars: 50 μm. Confocal microscopy analyses of CD3 (green), Pur-α (red), and DAPI in the lung tissues from OVA-immunized mice (**E**). Scale bars: 10 μm. OVA-specific T cell–mediated cytokine production (**F**). T cells were isolated from the lymph nodes of OVA-immunized mice on day 21 after immunization, followed by in vitro stimulation with OVA for 72 hours. The mRNA levels of *Il-9* and *Il-4* were determined using real-time PCR and were normalized to *Srp72* levels. *n* = 4 per group. WT (*Dusp8^fl/fl^*); T-*Dusp8* cKO, T cell–specific *Dusp8* cKO (*Dusp8^fl/fl^*;*Cd4*-*Cre*); cKO/*Pur-*α^+/–^, T-*Dusp8* cKO/*Pur-*α heterozygous KO mice; OVA, ovalbumin. Mean ± SEM are shown. *P value < 0.05; **P value < 0.01; ***P value < 0.001 (1-way ANOVA and Tukey’s posthoc test). *P* value in blue, WT versus cKO; *P* value in green, cKO/*Pur-*α^+/–^ versus cKO; NS, nonsignificant. Data shown are representative of 3 independent experiments.

Th9 cells contribute to allergic responses (9); therefore, we performed allergic asthma mouse models using T-*Dusp8*–cKO mice. Ovalbumin-stimulated (OVA-stimulated) inflammation in the lung tissues was induced in WT mice, whereas the lung inflammation was attenuated by T cell–specific *DUSP8* cKO (Figure 5A). To study whether DUSP8 mediates allergic responses through Pur-α, we also performed OVA model using T-*Dusp8* cKO/*Pur-*α heterozygous–KO mice, as *Pur-*α homozygous mice showed premature death. The attenuated lung inflammation in OVA-challenged T-*Dusp8*–cKO mice were indeed restored by *Pur-*α heterozygous KO (Figure 5A). Consistently, BALF IL-9 levels were significantly decreased in OVA-challenged T-*Dusp8*–cKO mice compared with those of WT mice (Figure 5B), while the reduction of BALF IL-9 levels in OVA-challenged T-*Dusp8*–cKO mice was reversed by *Pur-*α heterozygous KO (Figure 5B). Similarly, serum IgE levels were significantly decreased in OVA-challenged T-*Dusp8*–cKO mice, whereas IgE levels were restored in T-*Dusp8* cKO/*Pur-*α heterozygous–KO mice (Figure 5C). In contrast, the levels of Th2-cytokines IL-4, IL-5, IL-13, and IL-33 were only slightly decreased in OVA-challenged T-*Dusp8*–cKO mice (Figure 5B); the slight reduction of Th2 cytokines in T-*Dusp8*–cKO mice may be due to the secondary effect of Th9 reduction. In contrast, the BALF levels of the Th1 cytokine IFN-γ were unchanged in OVA-challenged T-*Dusp8*–cKO mice (Figure 5B). For the lung-infiltrating immune cells, the IL-9–producing T cells (Figure 5D and Supplemental Figure 11A), eosinophils (Supplemental Figure 11, B and D), and mast cells (Supplemental Figure 11, C and D) were decreased in the lung tissues of OVA-challenged T-*Dusp8*–cKO mice. Notably, the IL-9–producing T cells were mainly CD4^+^ T cells but not CD8^+^ T cells (Supplemental Figure 11A). In contrast, the reduction of Th9 cells, eosinophils, and mast cells in the lung tissues of T-*Dusp8*–cKO mice was restored by *Pur-*α heterozygous KO (Figure 5D and Supplemental Figure 11, A–D). Moreover, Pur-α localized to the cytoplasm of the infiltrating CD3^+^ T cells within the lung tissues of WT mice, whereas Pur-α mainly localized to the nucleus of the lung-infiltrating T cells of OVA-challenged T-*Dusp8*–cKO mice (Figure 5E). Interestingly, the signals of nuclear Pur-α were greatly decreased by *Pur-*α heterozygous KO (Figure 5E). Furthermore, in vitro OVA-restimulated *Il-9* induction in lymph node T cells was blocked by *DUSP8* cKO; in contrast, the *Il-9* reduction in *DUSP8* conditional–KO T cells was restored by *Pur-*α heterozygous KO (Figure 5F). Unlike *Il-9* induction, OVA-restimulated *Il-4* induction was modestly decreased by *DUSP8* cKO, and *Il-4* levels were not restored by *Pur-*α heterozygous KO (Figure 5F). These results suggest that DUSP8 intrinsically induces IL-9 production, but not IL-4 production, through Pur-α in OVA-immunized T cells. Nevertheless, we cannot rule the possibility that the DUSP8–Pur-α axis may also regulate other allergy-associated cytokines directly or indirectly. Collectively, DUSP8 blocks the suppressive function of Pur-α in T cells, contributing to T cell-mediated allergic responses.

### DUSP8 production and DUSP8–Pur-α interaction are induced in the cytoplasm of Th9 cells from people with asthma and patients with atopic dermatitis.

**Figure 6:**
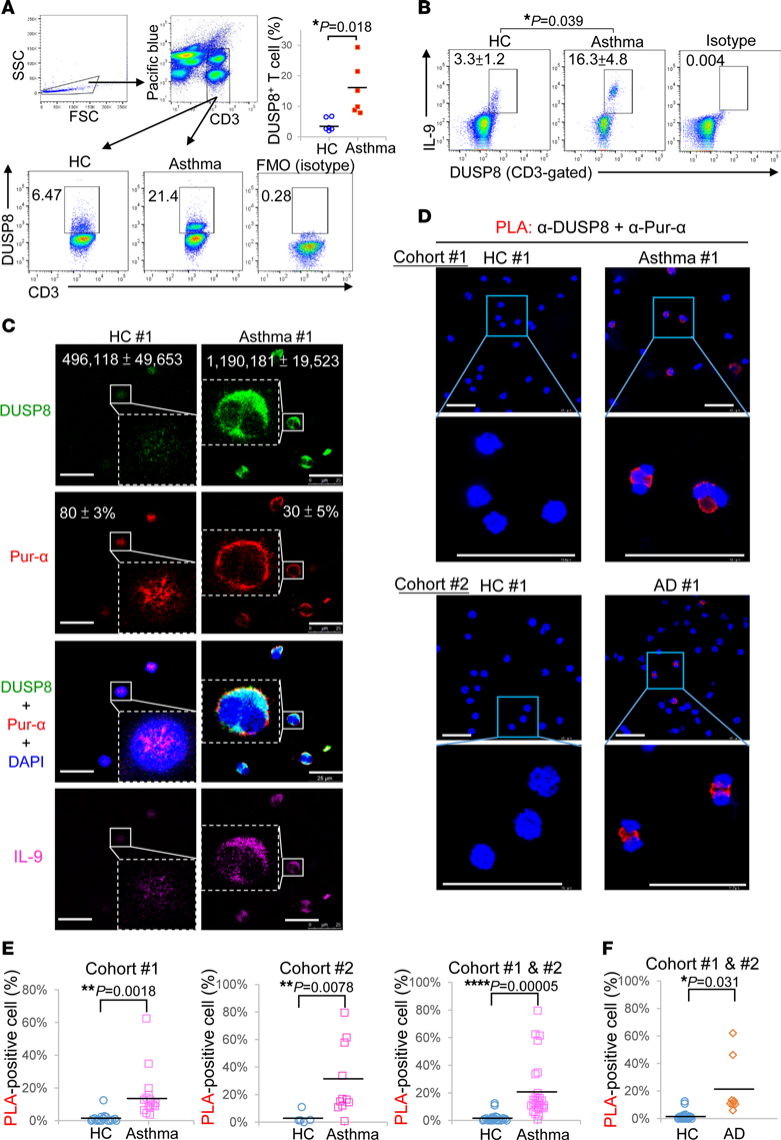
DUSP8 production and DUSP8–Pur-α interaction are induced in Th9 cells of people with asthma and atopic dermatitis. (**A**) Flow cytometry analyses of DUSP8-postivie T cells in the PBLs of 1 representative person from the healthy-control group (HC) and person with asthma. Data shown are mean ± SEM, *n* = 6 (right panel). There were no antibodies in Pacific Blue channel (left panel). FMO, fluorescence minus 1 (isotype control). (**B**) Flow cytometry analyses of DUSP8-postive and IL-9–producing T cells in the PBLs of a representative person from the healthy-control (HC) group and person with asthma. Data shown are mean ± SEM, *n* = 4. Isotype denotes the control group with isotype controls for anti-IL-9 and anti-DUSP8 antibodies. (**C**) Confocal microscopy of DUSP8, Pur-α, DAPI, and IL-9 in the peripheral blood T cells of 1 representative person from the HC group and person with asthma. Mean ± SEM of DUSP8 levels (green intensity) and Pur-α nuclear localization (red over blue, %) from 3 images are shown. Additional 11 people from the HC group, 23 people with asthma, and 7 people with atopic dermatitis (AD) are shown in Supplemental Figure 12. Scale bars: 25 μm. (**D**) Confocal microscopy of PLA for the DUSP8–Pur-α interaction in peripheral blood T cells, which were freshly isolated from 27 people with asthma and 18 people from the healthy-control group. One representative person from the HC group and person with asthma from Cohort 1 are shown (upper). One representative person from the HC group and person with AD from Cohort 2 are shown (lower). For PLA, red dots represent direct interaction signals. Scale bars: 50 μm. (**E**) Quantification plots of PLA signals (panel **D**) in asthma T cells from Cohort 1 (left), Cohort 2 (center), and the combination of 2 cohorts (right). (**F**) Quantification plots of PLA signals in AD T cells (panel **D**) from combination of 2 cohorts. *P < 0.05; **P < 0.01; ****P < 0.0001 (2-tailed Student’s *t* test).

**Figure 7:**
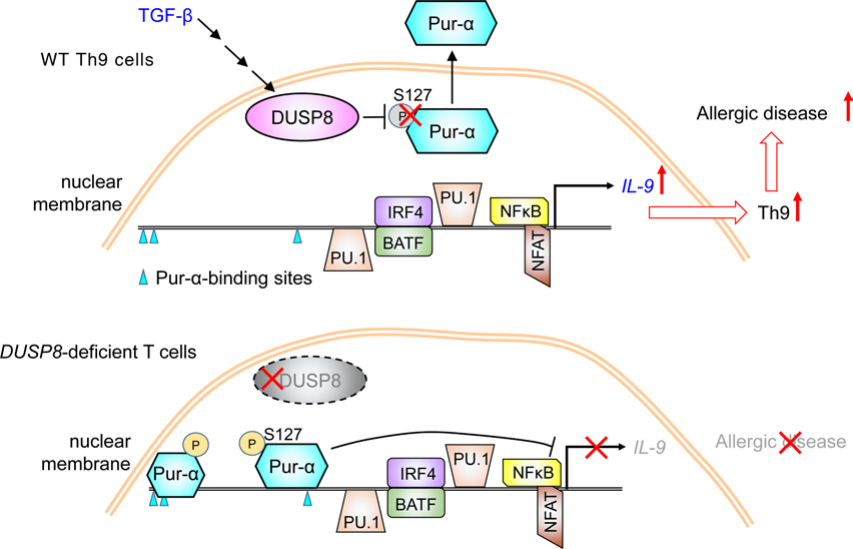
Schematic diagram for the induction of *IL-9* transcription by DUSP8. TGF-β signaling induces DUSP8 phosphatase activity. DUSP8 induces *IL-9* transcription by dephosphorylating the transcriptional repressor Pur-α in the nucleus. Dephosphorylated Pur-α is exported from the nucleus to the cytoplasm in T cells, resulting in induction of *IL-9* transcription. As a result, DUSP8 promotes Th9-mediated allergic diseases.

To study whether our findings are translatable to human allergic diseases, we enrolled people with allergic asthma and characterized their peripheral blood T cells (Supplemental Tables 1 and 2). To understand whether protein levels of DUSP8 or other DUSP family members are increased in asthma patients, protein lysates of freshly isolated peripheral blood T cells were digested by trypsin and subjected to mass spectrometry–based proteomics analysis. DUSP4, DUSP6, DUSP7, DUSP8, DUSP10, DUSP16, DUSP19, and DUSP21 were detectable in T cells from either individuals in the control group or patients with asthma (Table 1). Remarkably, DUSP8 was detected in 5 of 6 patients with asthma, whereas DUSP8 was detected in only 1 of 6 people in the control group (Table 1). Furthermore, the protein scores of DUSP8 were significantly increased in people with asthma compared with those of people in the control group (Table 1). Nevertheless, we could not rule out the possibility that additional DUSPs may be detected by proteomics analysis using peptidases other than the proteolytic enzyme trypsin. Consistent with the above mentioned results of animal studies, the flow cytometry analysis showed that the frequencies of DUSP8-positive T cells were increased in people with asthma compared with those of healthy controls (Figure 6A). The DUSP8 induction was concomitant with IL-9 production in patient T cells (Figure 6B). The induction of DUSP8 and IL-9 in the same T cells of people with asthma were also detected by immunofluorescence staining (Figure 6C and Supplemental Figure 12). As expected, DUSP8 proteins were colocalized with Pur-α in the cytoplasm of T cells from people with asthma (Figure 6C and Supplemental Figure 12). Moreover, in situ PLA showed that the interaction between DUSP8 and Pur-α was induced in the cytoplasm of the T cells from people with asthma of 2 independent cohorts (Figure 6D); the frequencies of the PLA-positive T cells were drastically increased in people with asthma compared with those of healthy controls (Figure 6E). To study whether the DUSP8–Pur-α–IL-9 axis contributes to other human allergic diseases, we further enrolled people with atopic dermatitis (AD). Interestingly, the induction of DUSP8 in the peripheral blood T cells of people with AD were also detected by immunofluorescence staining (Supplemental Figure 12). The colocalization of DUSP8 and Pur-α was detected predominantly in the cytoplasm of peripheral T cells from people with AD by immunofluorescence staining (Supplemental Figure 12). Furthermore, the DUSP8 and Pur-α interactions in the cytoplasm detected by PLA assays were also significantly induced in T cells of people with AD (Figure 6, D and F). These results suggest that DUSP8 interacts with the transcription repressor Pur-α and induces Pur-α nuclear export, resulting in IL-9 induction in the T cells of people with asthma or AD (Figure 7).

## Discussion

A key finding of this study was the identification of what we believe to be 2 novel IL-9 upstream regulators, DUSP8 and Pur-α. DUSP8 directly dephosphorylates the transcriptional repressor Pur-α at the Ser127 residue, resulting in nuclear export of the dephosphorylated Pur-α and subsequent induction of *IL-9* transcription. The complex of Pur-α and the Purα-binding protein (PurBP) translocates from the nucleus to the cytoplasm (24). Pur-α also regulates the subcellular localization of Rac1 and RhoA in developing neurons (25). To date, the mechanism of Pur-α nuclear export is unclear. In this report, our findings indicate that Ser127 phosphorylation controls Pur-α nuclear translocation, which is inhibited by DUSP8. *Il-9* mRNA levels in T cells were decreased in T-*Dusp8*–cKO mice, whereas the reduction of *Il-9* mRNA levels in T-*Dusp8*–cKO T cells was reversed by *Pur-*α heterozygous KO. Consistently, we examined the GEO Data set (GDS5343) (26) and found that *Pur-*α mRNA levels were inversely correlated with *Il-9* mRNA levels in differentiated murine Th9 cells under additional TCR stimulation, supporting the inhibitory role of Pur-α in controlling *Il-9* transcription. To our knowledge, this is the first report that Pur-α is a transcriptional repressor of *IL-9* transcription.

Pur-α is also a transcriptional repressor for other genes such as CD43 (27); in contrast, Pur-α is a transcriptional activator for *c-Myc* and *PCK2* (28). The dual roles of Pur-α in transcriptional activation or suppression may be due to its interaction with different proteins, its different posttranslational modifications, or different contexts of the Pur-α–binding sites within different promoters. Pur-α ChIP-Seq data derived from T-*Dusp8*–cKO T cells showed that, besides the *Il-9* promoter, Pur-α may also bind to other regions of the *Il-9* locus, leading to the inhibition of *Il-9* transcription. In addition, Pur-α also bound to multiple genes belonging to the pathways of mitophagy, cholesterol metabolism, TNF signaling, ubiquitin-mediated proteolysis, and protein processing (Supplemental Figure 6). Similarly, scRNA-Seq data of T-*Dusp8*–cKO T cells showed that DUSP8 is involved in the pathways of autophagy reactome, cellular metabolic process, cellular responses to stress, and protein folding (Figure 1D). Notably, ChIP-Seq data using T-*Dusp8*–cKO T cells showed that Pur-α targeted signaling molecules of mTOR signaling and HIF-1α signaling (Supplemental Figure 6) which also regulate Th9 generation (29) and T-cell mediated immune responses (30). Collectively, DUSP8 induces IL-9 production and may also regulate other genes/pathways by inhibiting Pur-α function.

An exciting finding in this report is that DUSP8 plays a critical role in Th9-mediated allergic diseases. Our data showed that the induction of asthma phenotypes in the mouse model was attenuated by T-cell-specific *DUSP8* cKO. Consistently, among 25 phosphatases of the DUSP phosphatase family, only DUSP8 proteins in T cells of people with asthma were induced, detected by mass spectrometry-based proteomics. Furthermore, DUSP8 induction in T cells also occurred in T cells of people with atopic dermatitis. The concomitant induction of DUSP8 and IL-9 in peripheral blood T cells of people with asthma and AD supports that DUSP8 induces IL-9 production. Moreover, the nuclear export of Pur-α and the cytoplasmic interaction between DUSP8 and Pur-α also occurred in the peripheral blood T cells of people with asthma and atopic dermatitis. These results suggest that DUSP8 interacts with Pur-α and induces Pur-α nuclear export in T cells, leading to the inhibition of Pur-α suppressive function and the induction of *IL-9* transcription (Figure 7). Interestingly, the *DUSP8* SNP table in GeneCards (Human Gene Database) website shows that a SNP (rs61867538) within the DUSP8 gene is associated with *IL-9* levels (www.genecards.org/cgi-bin/carddisp.pl?gene=DUSP8). Moreover, 2 other SNPs (rs17885785 and rs1004446), mapped to the putative enhancers of the DUSP8 gene by GeneHancer database of the GeneCards website, are associated with several autoimmune diseases, including systemic lupus erythematosus, chronic childhood arthritis, psoriasis, ulcerative colitis, and type I diabetes mellitus. Our findings and the database information support that DUSP8 is a key activator of IL-9 production and may be involved in human allergic diseases. In addition, 1 SNP (rs11554776), mapped to the putative enhancers of the Pur-α gene by the GeneHancer database, is also associated with psoriasis. It would be valuable to explore whether DUSP8 and Pur-α also play an important role in the pathogenesis of autoimmune diseases. Taken together, our findings suggest that DUSP8 is a T cell biomarker and potential therapeutic target for IL-9-mediated allergic diseases.

## Methods

### Study participants.

This study was conducted in accordance with the Helsinki Declaration. A total of 58 individuals, including 23 people who were healthy, 27 people with asthma, and 8 people with AD were enrolled in this study. For Cohort 1, 18 people in the control group, 16 people with asthma, and 6 people with AD were referred to National Taiwan University Hsin-Chu Hospital in Taiwan (Supplemental Table 1). For Cohort 2, 5 people in the control group, 11 people with asthma, and 2 people with AD were referred to the Division of Immunology and Rheumatology at Taipei Veterans General Hospital or Taichung Veterans General Hospital in Taiwan (Supplemental Table 2).

### Mice.

All animal experiments were performed in the Association for Assessment and Accreditation of Laboratory Animal Care–accredited (AAALAC-accredited) animal housing facilities according to the protocols and guidelines approved by the IACUC of National Health Research Institutes (NHRI). A C57BL/6J mouse embryonic stem cell clone (ID 25378, clone EPD0754_2_D07) with *Dusp8* KO-first allele from the Knockout Mouse Project (KOMP) was injected into C57BL/6J blastocysts to generate chimeric mice by NHRI Transgenic Mouse Core. The KO-first allele is initially a nonexpressing state, but can be converted to a conditional floxed allele via Flp recombination using *Actin-Flp* transgenic mice (JAX 003800). T-*Dusp8*–cKO mice were generated by crossing floxed *Dusp8* mice and *Cd4-Cre* transgenic mice (JAX 022071). *Pur-*α–KO mice in C57BL/6J background were generated using the CRISPR/Cas9 approach by NHRI Transgenic Mouse Core. Thirty-four nucleotides encompassing the start codon (ATG) in the *Pur-*α exon 2 were deleted in the mutated allele. In this study, sex-matched, 3-to-24 week-old littermate mice were used for experiments. All mice were maintained in temperature-controlled and pathogen-free cages.

### Allergic asthma model.

For the induction of asthma model, 8-to-10 week-old mice were sensitized by i.p. injection of 10 mg ovalbumin (OVA; Sigma-Aldrich) adsorbed with 200 μL aluminum hydroxide (Sigma-Aldrich) on day 0 and day 14. From day 21, mice were exposed to aerosolized OVA (1% wt/vol) for 30 min/day for 5 consecutive days. To collect the lung-infiltrating immune cells from OVA-immunized mice, lung tissues were homogenized by gentleMACS Dissociator (Miltenyi Biotec), followed by incubation with RPMI media containing collagenase II (Sigma-Aldrich) at 37 °C for 15 minutes. After washing with 15 mL PBS, the infiltrating immune cells were isolated by Percoll (Cytiva) gradient centrifugation. The isolated immune cells were subjected to flow cytometry analysis for Th9 cells (CD45^+^, CD3^+^, CD4^+^, and IL-9^+^), IL-9-producing CD8^+^ T cells (CD45^+^, CD3^+^, CD8^+^, and IL-9^+^), eosinophils (CD45^+^, Ly6G^–^, CD11b^+^, and CD11c^–^) (12), and mast cells (CD45^+^, CD49b^–^, c-kit^+^, and FcεR1^+^) (12). For detection of IL-9-producing T cells, immune cells were incubated in MACS buffer containing anti-mCD45 (130-110-665, Miltenyi Biotec), anti-mCD3 (553067, BD Biosciences), anti-mCD4 (558107, BD Biosciences), and anti-mCD8 (557654, BD Biosciences) antibodies plus GolgiStop (1:1,000, BDB554724, BD Biosciences) for 3 hours at room temperature without any stimulation, followed by incubation in 200 μL Cytofix/Cytoperm buffer (BD Biosciences) at 4 °C overnight. For OVA-specific T-cell responses, T cells were isolated from draining lymph nodes of individual mice on day 21 after immunization. 5 × 10^6^ T cells and 5,000 WT B cells were cocultured in RPMI media containing OVA (0, 20, or 50 μg/mL) for 72 hours.

### Plasmids.

The plasmid-expressing 3xFlag-tagged human *Pur-*α cDNA (NCBI accession number: NM_008989) was generated by subcloning *Pur-*α cDNA into the vector pCMV6-AN-3DDK (OriGene). The plasmid encoding Myc-tagged mouse DUSP8 was generated by cloning the *Dusp8* cDNA into the vector pMTSM-Myc (6). The plasmid expressing DUSP8 (C246S) phosphatase–dead mutant was generated from the Myc-*DUSP8* plasmid by mutating cysteine-246 to serine using PCR-based site-directed mutagenesis. The plasmids expressing YFP-fused DUSP8 and CFP-fused Pur-α proteins were constructed by subcloning the *Dusp8* cDNA and *Pur-*α cDNA from the Myc-*Dusp8* plasmid and 3xFlag-*Pur-*α plasmid into the pCMV6-AC-*Yfp* vector (OriGene) and pCMV6-AN-*Cfp* vector (OriGene), respectively. The plasmid expressing GFP-fused Pur-α protein was generated by subcloning the human *Pur-*α cDNA into the vector pCMV6-AN-*Gfp* (OriGene). The plasmids encoding Pur-α (S217A) and Pur-α (T183A) mutant proteins were generated by mutating the indicated amino acid residues in the 3xFlag-*Pur-*α or GFP-*Pur-*α plasmid.

### Reagents and antibodies.

The rabbit antibody recognizes both human and murine DUSP8 proteins was generated by immunization of a rabbit with peptides (murine DUSP8 epitope: ^268^SSDDAYRFVKDRRPSISPN^286^; corresponding to the human DUSP8 protein sequences ^268^SSDDAYRFVKDRRPSISPN^286^). Murine anti-Pur-α antibody (clone 1C10; AT3501a) and rabbit anti-Pur-α antibody (ab125200) were purchased from Abcepta and Abcam, respectively. Alexa 594-conjugated donkey anti-rabbit IgG (ab150064) and Alexa 488-conjugated donkey anti-mouse IgG (ab150105) antibodies were purchased from Abcam. Anti-mCD3-PerCP (clone 145-2C11, 553067), anti-mCD4-pacific blue (clone RM4-5, 558107), anti-mCD8-APC-Cy7 (clone 53-6.7, 557654), anti-mIL-17A-Alexa 647 (clone TC11-18H1, 560184), anti-mFcεR1-Alexa 488 (clone MAR-1, 567801), anti-mCD49b-APC (clone DX5, 560628), anti-c-Kit-PerCP-Cy5.5 (clone2B8, 560557), anti-hCD3-PE-Cy7 (clone SK7, 557851), and anti-hIL-9-PerCP-Cy5.5 (clone MH9A3, 561461) antibodies were purchased from BD Biosciences. Anti-Foxp3-PE (clone 150D, 320008), anti-mIL-4-PE (clone 11B11, 504104), anti-mIL-9-PE (clone RM9A4, 514104), anti-mIFN-γ-FITC (clone XMG1.2, 505806), anti-mLy6G-pacific blue (clone 1A8, 127612), anti-mCD11c-APC (clone N418, 117310), and anti-mCD11b-PerCP-Cy5.5 (clone M1/70, 101228) antibodies were purchased from BioLegend. Anti-mCD45-VioGreen antibody (clone REA737, 130-110-665) was purchased from Miltenyi Biotec. Fixable Viability Stain 780 reagent was purchased from BD Biosciences. Opal 4-color manual IHC kit was purchased from PerkinElmer. Anti-mast cell tryptase (GTX32931) and anti-PRG2 (GTX54611) were purchased from GeneTex. The mouse cytokine array kit (Proteome Profiler Array, #ARY0028) was purchased from R&D. ELISA kits of mouse IL-2 and IFN-γ were purchased from Thermo Fisher Scientific. ELISA kits for mouse IL-4, IL-5, IL-9, IL-13, and IL-33 were purchased from R&D Systems. The ELISA kit for mouse IL-17A was purchased from BioLegend. For detection of *Pur-*α mRNA levels by Q-PCR, the primer pairs and probes (Mm01158049) were purchased from Thermo Fisher Scientific.

### T cell purification and in vitro T cell differentiation using murine primary T cells.

Murine T cell purification and in vitro Th1/Th2/Th17/Treg differentiation assays were performed using the methods described previously (31–33). For Th9 differentiation, 5 × 10^5^ splenic CD4^+^ T cells were cultured for 5 days in 1 mL Iscove’s Modified Dulbecco’s Media (IMDM) in 48-well plates coated with anti-CD3 (2.5 μg) and anti-CD28 (2.5 μg) antibodies. The IMDM media contained TGF-β (10 ng/mL), mIL-4 (20 ng/mL), mIL-2 (20 ng/mL), and anti-IFN-γ antibodies (10 μg/mL). Before harvesting cells, murine T cells were stimulated for 4 hours with 0.1% PMA and 0.1% ionomycin in the presence of 0.1% GolgiStop reagent (BD Biosciences).

### Human primary Th9-cell polarization and cell transfection.

Human CD4^+^ T cells were freshly isolated by negative selection from peripheral blood leukocytes (PBLs) of healthy volunteers. 5 × 10^5^ splenic CD4^+^ T cells were cultured for 5 days in 1 mL Iscove’s Modified Dulbecco’s Media (IMDM) in 48-well plates coated with anti-CD3 (2.5 μg) and anti-CD28 (2.5 μg) antibodies. The IMDM media contained TGF-β (10 ng/mL), mIL-4 (20 ng/mL), mIL-2 (20 ng/mL), and anti-IFN-γ antibodies (10 μg/mL). Differentiated Th9 cells were transfected with 15 μg GFP-fusion *Dusp8* plasmid, DUSP8 shRNA within *Gfp*-expressing plasmid, or GFP-fusion *Pur-*α plasmid by Neon electroporator (Invitrogen). The electroporation condition was 2,000 V, 20 ms duration, and 1 pulse. After 30 hours, the transfected T cells were sorted by Influx cell sorter (BD Biosciences, Supplemental Figure 4C) and subjected to real-time PCR.

### Flow cytometry using human PBLs.

Human PBLs were freshly isolated from 10 mL whole blood by centrifugation, followed by washing twice with 50 mL ACK (ammonium-chloridepotassium) buffer. 1 × 10^6^ PBLs were first incubated in MACS buffer containing anti-hCD3-PE-Cy7 (1:100, BD Biosciences) and GolgiStop (1:1,000, BD Biosciences) for 3 hours at room temperature without any other stimulation (32). The PBLs were incubated in 200 μL Cytofix/Cytoperm buffer (BD Biosciences) at 4 °C overnight. The permeabilized cells were washed with Perm/Wash buffer (BD Biosciences) and then incubated in the Perm/Wash buffer containing anti-DUSP8 antibody (1:500) and anti-hIL-9-PerCP-Cy5.5 (1:50) at 4 °C for 2 hours, followed by staining with anti-rabbit IgG-FITC (1:400) for 1 hour. After washing with MACS buffer, the cells were detected by FACSCanto II flow cytometer (BD Biosciences). Notably, when we processed clinical samples using a previously published method (34), the frequency of IL-9-producing T cells in an asthma patient shown in Figure 6B would be decreased from 10.2% to 1.17%. It is likely that the incubation time for GolgiStop and permeabilization is critical.

### scRNA-Seq.

Murine T cells were isolated by negative selection from the spleen and lymph nodes of T-*Dusp8*–cKO and WT mice. CD4^+^ T cells and CD8^+^ T cells were labelled by the AbSeq anti-CD4 and anti-CD8 antibody-oligonucleotide conjugates (BD Biosciences), respectively. scRNA-Seq was performed using BD Rhapsody Single-Cell Analysis System (BD Biosciences). The scRNA-Seq data were analyzed as described previously (35–37); scRNA-Seq data are available in a public, open access, NIH repository (SRA no. SRR25734299).

### Luciferase reporter assays of the Il-9 promoter.

Luciferase reporter assays for the mouse *Il-9* promoter activity were performed using the Secrete-Pair Dual Luminescence Assay kit (GeneCopoeia) according to the manufacturer’s instructions as described previously (38). 5 × 10^6^ Jurkat T cells were transfected with the 1,123-bp (–1,097 to +26) *Il-9* promoter-driven gaussia-luciferase reporter plasmid, which contained the cDNA sequences encoding the secreted alkaline phosphatase (SEAP). Forty-eight hours after transfection, T cells were stimulated with TGF-β (10 ng/mL) for 6 hours. For the detection of gaussia luciferase levels, 10 μL T cell supernatants were incubated with 100 μL buffer containing the gaussia luciferase substrate for 30 seconds. For the detection of SEAP levels, 10 μL supernatants were incubated at 65 °C for 5 minutes, followed by incubating with the SEAP substrate for 5 minutes. The gaussia luciferase levels were normalized to the SEAP levels.

### ChIP-Seq.

T cells were isolated from the spleen and lymph nodes of T-*Dusp8*–cKO or WT mice. ChIP was performed using the Pierce Magnetic ChIP kit (Thermo Fisher Scientific) according to the manufacturer’s instructions. Briefly, T cells were cross-linked with 1% formaldehyde at 37 °C for 10 minutes. To generate DNA fragments, the T cell lysates were sonicated (3 times for 9 seconds each time) on ice. Cell extracts were immunoprecipitated with protein G-conjugated Dynabeads (Invitrogen) plus anti-Pur-α antibody (5 μg, ab125200, Abcam) at 4 °C on a rotating wheel for 4 hours. To reverse cross linking, Pur-α immunocomplexes were incubated at 95 °C for 15 minutes, followed by proteinase K treatment. Immunoprecipitated DNA fragments were eluted using PCR purification kit (GE Healthcare) and subjected to Illumina Next-Generation Sequencing (NGS). ChIP-Seq data are available in a public, open access repository (SRA #PRJNA1008381).

### Immunofluorescence staining and confocal microscopy.

Human peripheral blood T cells or murine T cells were fixed with cold methanol for 2 minutes, followed by permeabilization using 0.1% Triton X-100 for 3 hours. The fixed T cells were incubated with antibodies against DUSP8 (1:200), Pur-α (1:200, clone 1C10, Abcepta), BATF (1:200, 8638, Cell Signaling Technology), PU.1 (1:500, MA5-15064, Invitrogen), or IRF-4 (1:100, 4964, Cell Signaling Technology) at 4 °C for overnight, followed by incubation with Alexa Fluor-488 anti-rabbit IgG (1:400, ab150061, Abcam) and Alexa Fluor-594 anti-mouse IgG (1:400, ab150108, Abcam) antibodies for 1 hour. For detection of IL-9, the stained cells were further incubated with anti-hIL-9-PerCP-Cy5.5 (1:100, clone MH9A3, BD Biosciences) antibody for 2 hours. Cell nucleus was stained with DAPI. DAPI, 4′,6-diamidino-2-phenylindole. Confocal images were acquired by Leica TCS SP5 II confocal microscope.

### Protein-protein interaction analyses.

The interaction between DUSP8 protein and Pur-α protein was determined by coimmunoprecipitation, FRET (detecting protein-protein interaction < 10 nm) (39), and in situ PLA, as described previously (37, 40).

### In situ PLA technology.

In situ PLA assays were performed using the duolink in situ-red kit (Sigma-Aldrich) according to the manufacturer’s instructions, as described previously (37, 40). Briefly, human peripheral blood T cells or murine T cells were fixed with cold methanol for 2 minutes, followed by permeabilization using 0.1% Triton X-100 for 3 hours. The methanol-fixed T cells were incubated with antibodies (1:500) against DUSP8 and Pur-α at 4 °C for overnight, followed by incubation with species-specific secondary antibodies conjugated with oligonucleotides (PLA probes, Sigma-Aldrich) at 37 °C for 1 hour. After ligation at 37 °C for 30 minutes, the PLA signals were amplified in the amplification buffer at 37 °C for 100 minutes. PLA signals from each pair of PLA probes in close proximity (< 40 nm; the DUSP8–Pur-α interaction) (41) were visualized as individual red dots and analyzed by Leica TCS SP5 II confocal microscope. Red dots represent direct interaction signals.

### In vitro DUSP8 phosphatase assays.

Myc-tagged DUSP8 proteins, Myc-tagged DUSP8 (C246S) phosphatase-dead mutant proteins, and Flag-tagged Pur-α proteins were immunoprecipitated from lysates of HEK293T transfectants, followed by peptide-elusion purification using Myc or Flag peptides. Purified Pur-α proteins were incubated with purified DUSP8 WT or mutant (C246S) proteins at 37 °C for 2 hours in the phosphatase reaction buffer (50 mM imidazole and 10 mM DTT). The reaction products were incubated in sample buffer for 3 minutes at 95 °C, followed by immunoblotting with anti-pan-phospho-serine (clone 4A4; Merck) and anti-pan-phospho-threonine (clone 42H4; Cell Signaling) antibodies.

### Liquid chromatography-mass spectrometry analysis.

To identify DUSP8-interacting proteins, proteins of the DUSP8 immunocomplexes were digested with trypsin and subjected to LC-MS/MS analyses by LTQ-Orbitrap Elite hybrid mass spectrometer, as described previously (37, 40). For the identification of DUSP8-dephosphorylated residues on Pur-α, Flag-tagged Pur-α proteins were isolated from the reaction mixtures of in vitro DUSP8 phosphatase assays, followed by SDS-PAGE fractionation and trypsin digestion. The trypsin-digested Pur-α peptides were subjected to LC-MS/MS analyses.

To characterize DUSP proteins in peripheral blood T cells of asthma patients and healthy controls, freshly isolated T cells without any stimulation were lysed with RIPA buffer, followed by SDS-PAGE fractionation and trypsin digestion. The digested peptides were subjected to LC-MS/MS analyses by LTQ-Orbitrap Elite hybrid mass spectrometer. The peptide data were analyzed by MASCOT MS/MS Ions Search (Matrix Science) under the following condition: peptide mass tolerance, 20 ppm; fragment MS/MS tolerance, 0.6 Da; allow up to 1 missed cleavage; peptide charge, 2^+^, 3^+^, and 4^+^. Mass spectrometry data are available in a public, open access repository (ProteomeXchange nos. PXD044818, PXD044898, and PXD044824).

### Statistics.

Statistical analyses were performed by using Excel 2016, SPSS 25, or BD SEQGEQ. Two groups were compared by 2-tailed or 1-tailed unpaired Student’s *t* tests, as well as Wilcoxon’s rank-sum test. Three or multiple groups were compared by ANOVA test, followed by Tukey’s posthoc test. *P* values under 0.05 were considered to be significant. Quantification of immunofluorescence or histological image was performed using ImageJ software.

### Study approval.

All animal experiments were approved by the IACUC of National Health Research Institutes (NHRI). All experiments using human clinical samples were approved by the Institutional Review Boards. Written informed consent (approved by the IRB at National Taiwan University Hsin-Chu Hospital, Taiwan [no. 109-008-E], Taichung Veterans General Hospital, Taiwan [no. SE17193B], or Taipei Veterans General Hospital, Taiwan [no. 2017-06-003BC]) was obtained from individual patients before enrollment in this study. All 3 Institutional Review Boards approved the experiments.

### Data availability.

ChIP-Seq data, scRNA-Seq data, and mass spectrometry data are available in a public, open access repository (SRA no. SRR25737105, SRA no. SRR25734299, ProteomeXchange no. PXD044818, ProteomeXchange no. PXD044898, and ProteomeXchange no. PXD044824). Other data are available upon reasonable request. Values for all data points in graphs are reported in the Supporting Data Values file.

## Author contributions

HCC performed and supervised experiments, data analysis, data interpretation, study design, and manuscript writing. CHH, PMH, CYT, and YCS performed experiments. HYC, YMC, WKY, and MHC provided patient samples and analyzed clinical data. THT conceived the study, supervised experiments, interpreted data, and wrote the manuscript.

## Supplementary Material

Supplemental data

Supporting data values

## Figures and Tables

**Table 1:**
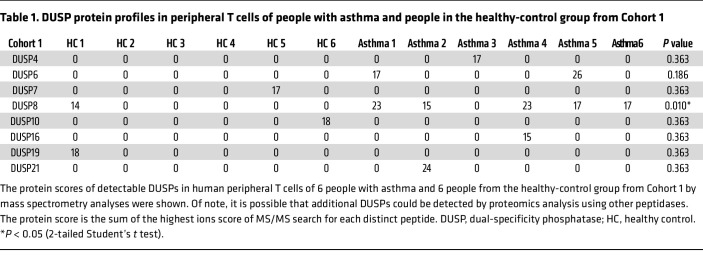
DUSP protein profiles in peripheral T cells of people with asthma and people in the healthy-control group from Cohort 1

## Version 1 11/01/2023

Electronic publication
