# Supplementary material for: DUSP8 induces TGF-β–stimulated IL-9 transcription and Th9-mediated allergic inflammation by promoting nuclear export of Pur-α
Source: J Clin Invest. 2023 Nov 1;133(21):e166269. doi: 10.1172/JCI166269 (PMC10617771; doi:10.1172/JCI166269)
Supplement: Supplemental data [file jci-133-166269-s020.pdf]

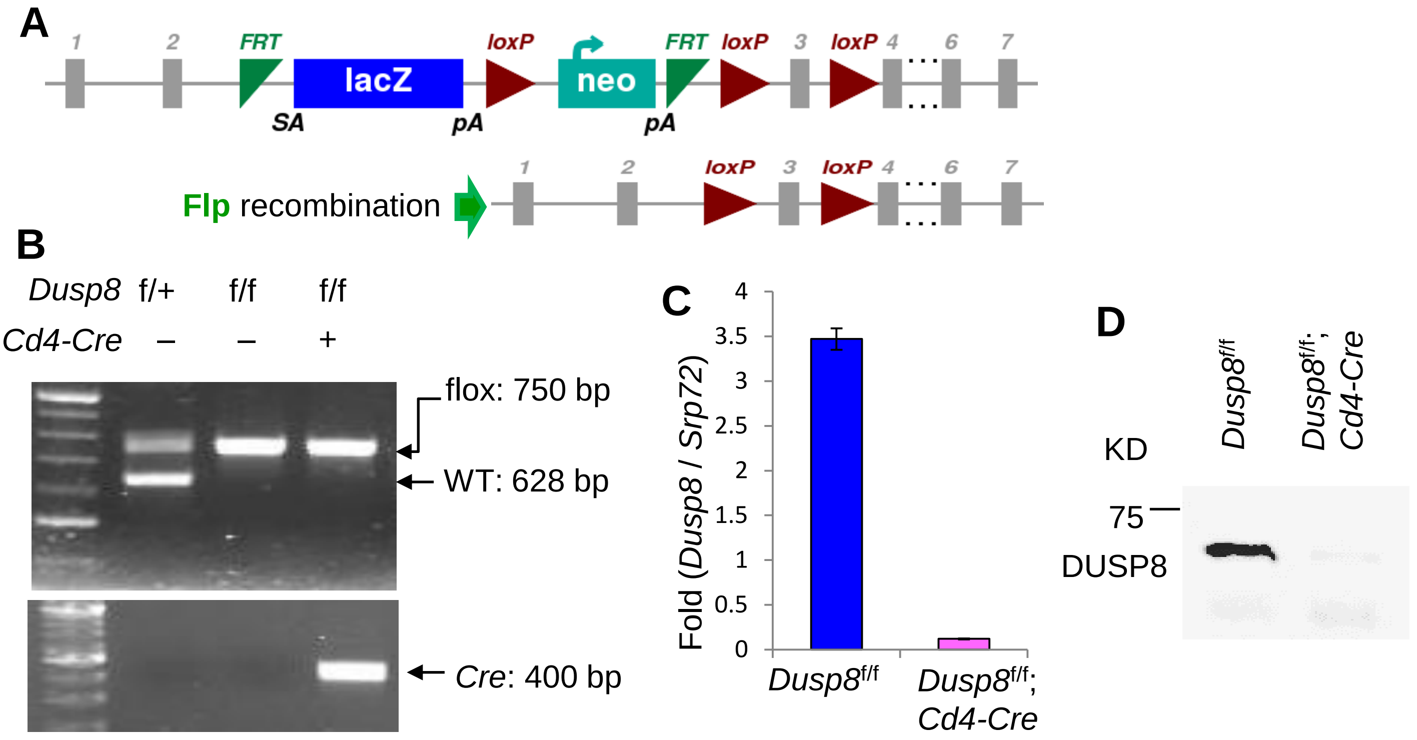

Supplemental Figure 1. Generation of T-cell-specific *Dusp8* conditional knockout mice. (**A**) The schematic diagram of the *Dusp8* knockout (KO) first allele (reporter-tagged insertion with conditional potential) and the resulting floxed *Dusp8* allele. An insertion vector containing a splice acceptor (SA), a fusion of *lacZ* and *neomycin resistance gene* (neo), and a polyadenylation (pA) sequence was inserted in the intron between exons 2 and 3 of the targeted *Dusp8* allele. The KO-first allele is initially a non-expressive form, but can be converted to a conditional floxed allele via Flp recombination. (**B**) The PCR product of the 750-bp band indicates the floxed *Dusp8* allele; the 628-bp band indicates the WT allele. Genomic DNAs were extracted from the tail of mice. (**C**) Real-time PCR analyses of *Dusp8* mRNA levels in peripheral blood T cells from 5 mice per group. *Dusp8* mRNA levels were normalized to *Srp72* levels. Means ± SD are shown. (**D**) T-cell-specific deletion of *Dusp8* in T-*Dusp8* cKO mice. Immunoblotting analysis of DUSP8 protein levels in purified peripheral T from T-*Dusp8* cKO (*Dusp8*$^{f/f}$;*Cd4-Cre*) and WT (*Dusp8*$^{f/f}$) mice. Data shown are representative of three independent experiments.

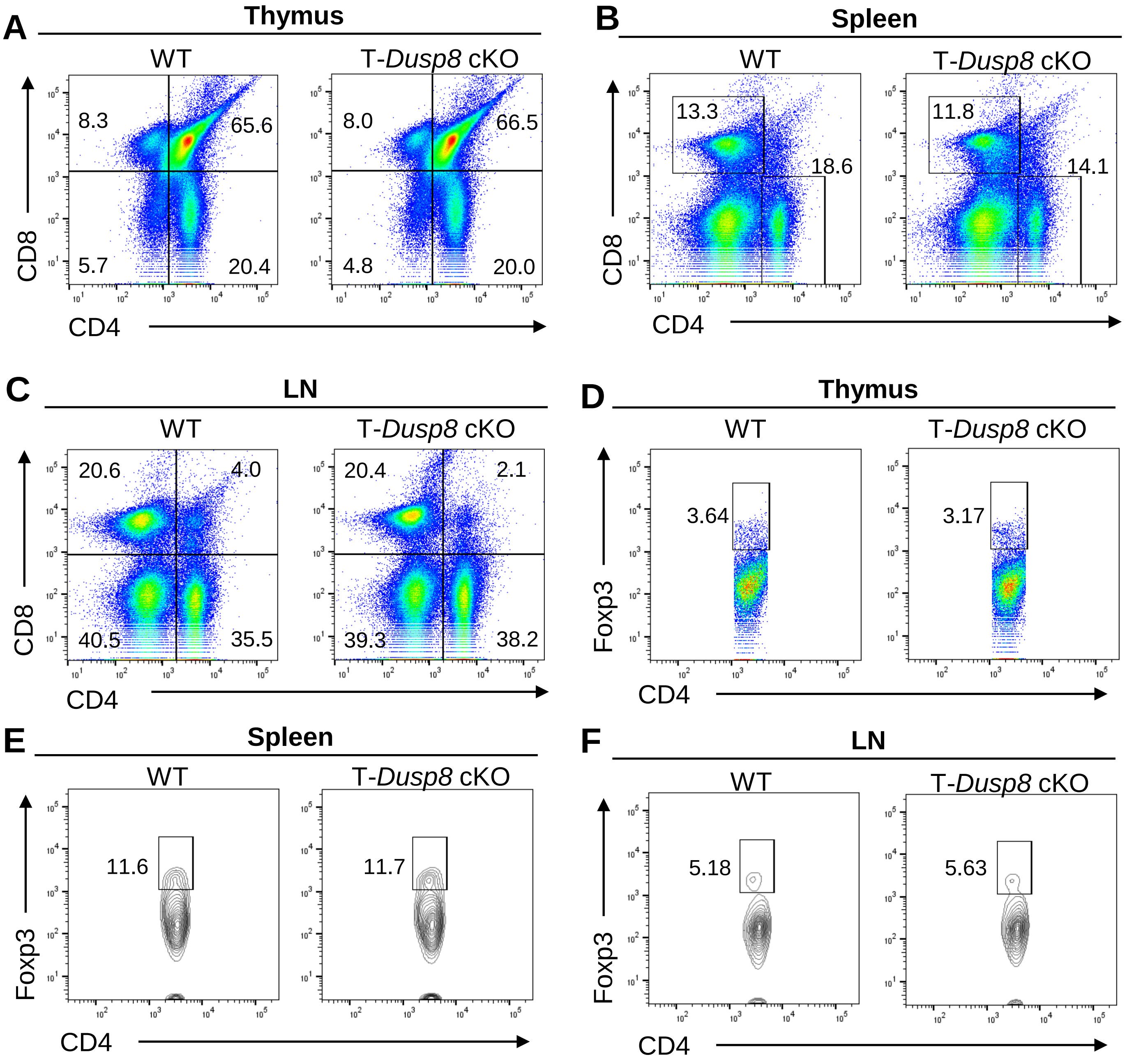

Supplemental Figure 2. T-*Dusp8* cKO mice display normal T-cell development. (**A-F**) Flow cytometry analyses of T cells (A-C) and Treg cells (D-F) from the thymus, spleen, or lymph nodes of 5-week-old T-*Dusp8* cKO or wild-type mice. T-*Dusp8* cKO, T-cell-specific *Dusp8* conditional knockout (*Dusp8*$^{f/f}$;*Cd4-Cre*); WT, wild-type (*Dusp8*$^{f/f}$). Data shown are representative of three independent experiments.

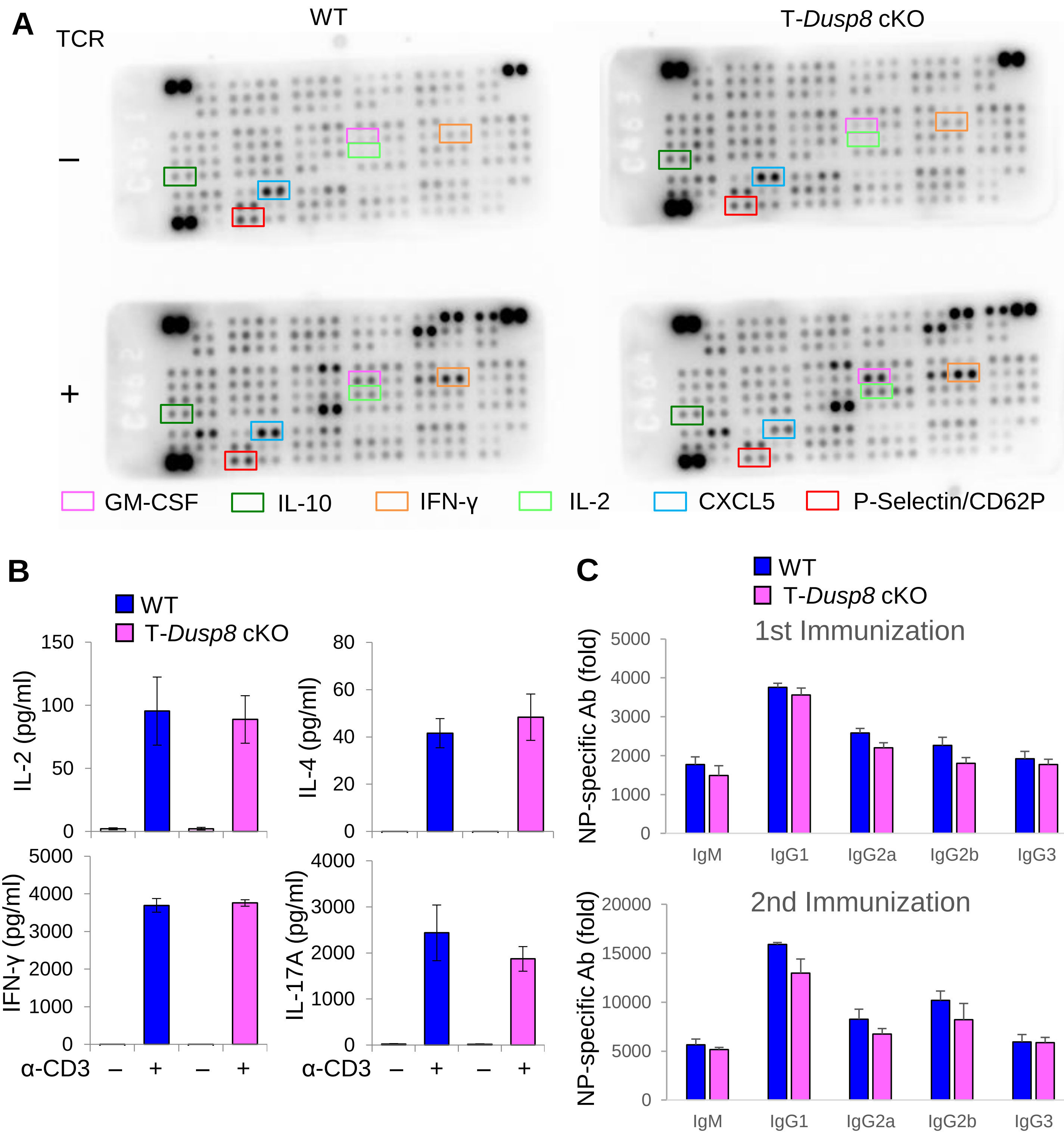

Supplemental Figure 3. T-cell receptor-stimulated cytokine levels of T-*Dusp8* cKO T cells are comparable to those of wild-type T cells. (**A**) Cytokine arrays of 111 cytokines shows individual cytokine levels in the supernatants of T cells from T-*Dusp8* cKO or wild-type mice. T cells were stimulated with anti-CD3 antibody for 72 h. Color frames denote cytokines that are slightly dysregulated in T-*Dusp8* cKO T cells compared to those of wild-type T cells. T-*Dusp8* cKO, *Dusp8*$^{f/f}$;*Cd4-Cre* mice; WT, *Dusp8*$^{f/f}$ mice; TCR, T-cell receptor signaling. (**B**) ELISA of IL-2, IL-4, IL-17A, and IFN-γ levels in the supernatants of anti-CD3-stimulated T cells from T-*Dusp8* cKO and wild-type mice. n = 6 (biological replicates). Means ± SEM are shown. (**C**) ELISA of serum nitrophenol-specific antibody (NP-specific Ab) of T-*Dusp8* cKO and wild-type mice. The sera were collected from immunized mice 14 days after primary immunization (upper panel) or 7 days after secondary immunization (lower panel). Means ± SD are shown. WT, n = 5; T-*Dusp8* cKO, n = 3 (biological replicates). Data are presented relative to those of normal serum from a wild-type mouse.

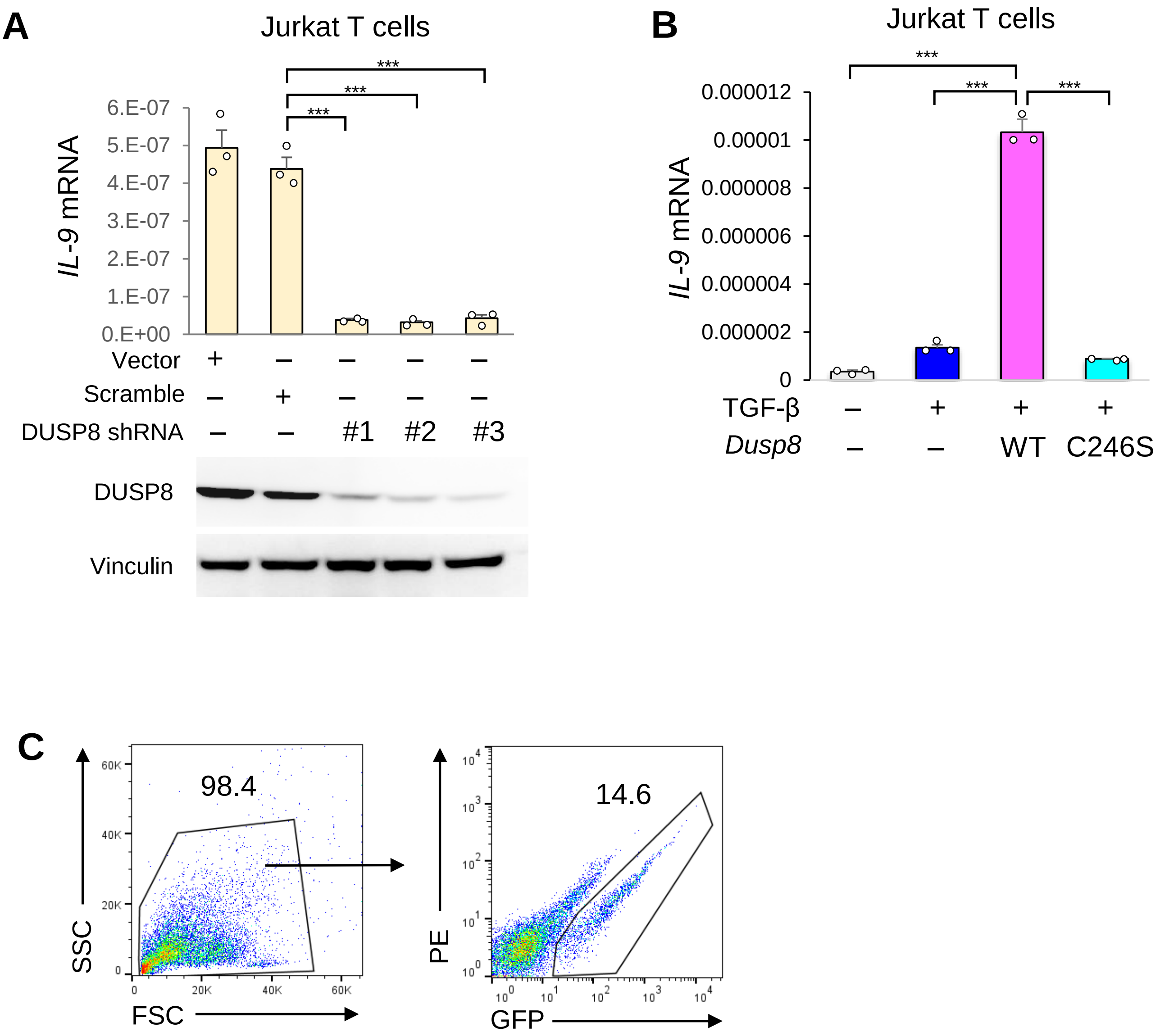

Supplemental Figure 4. DUSP8 positively regulates *IL-9* mRNA levels in Jurkat T cells stimulated with TGF-β. (**A** and **B**) Real-time PCR of *IL-9* mRNA levels in DUSP8 shRNA knocked-down Jurkat T cells (A) or DUSP8-overexpressing Jurkat T cells (B). Cells were stimulated with TGF-β (50 ng/ml) for 8 h. The expression levels of *IL-9* were normalized to *GAPDH* levels. Means ± SEM are shown. n = 3. DUSP8 protein levels in DUSP8 shRNA knocked-down Jurkat T cells were determined by immunoblotting analysis (A, lower panel). WT, wild-type; C246S, phosphatase-dead mutant. (**C**) Gating strategy for the transfected (GFP-positive) human primary Th9 cells by Influx cell sorter. ***, *P* value < 0.001 (one-way ANOVA and Tukey's post hoc test).

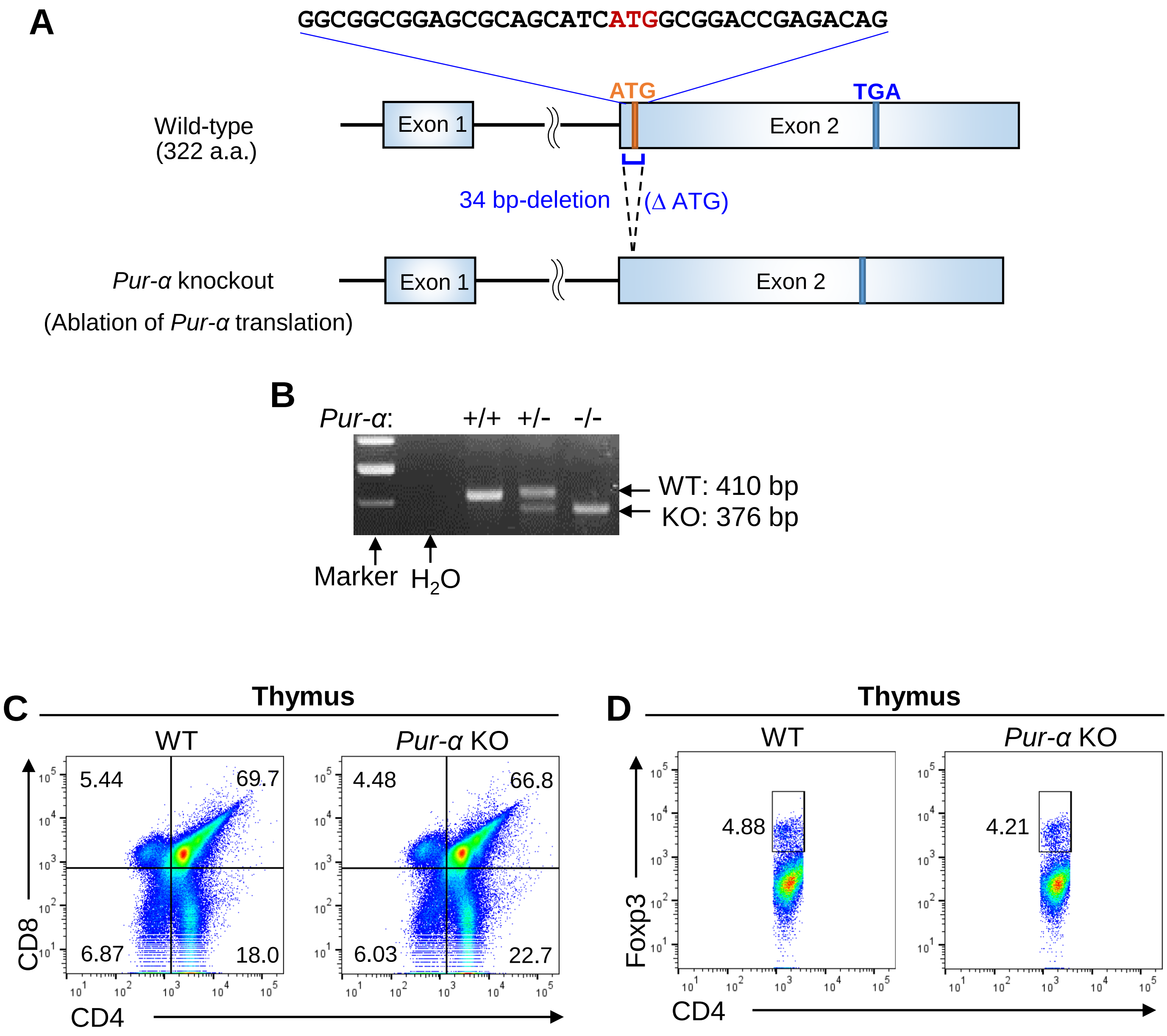

Supplemental Figure 5. *Pur-α* KO mice display normal T-cell development. (**A**) Schematic diagram of the murine *Pur-α* wild-type allele and the targeted *Pur-α* mutant allele. *Pur-α* knockout mice were generated by CRISPR/Cas9-mediated gene targeting. The deletion of 34 bp including the start codon (ATG) on exon 2 resulted in the obliterating of *Pur-α* translation. (**B**) The PCR product of the 410-bp band indicates the wild-type (WT) *Pur-α* allele; the 376-bp band indicates the *Pur-α* knockout allele. (**C** and **D**) Flow cytometry analyses of T cells (C) and Treg cells (D) from the thymus of 21-day-old *Pur-α* knockout or wild-type mice. WT, wild-type mice; KO, *Pur-α* knockout mice.

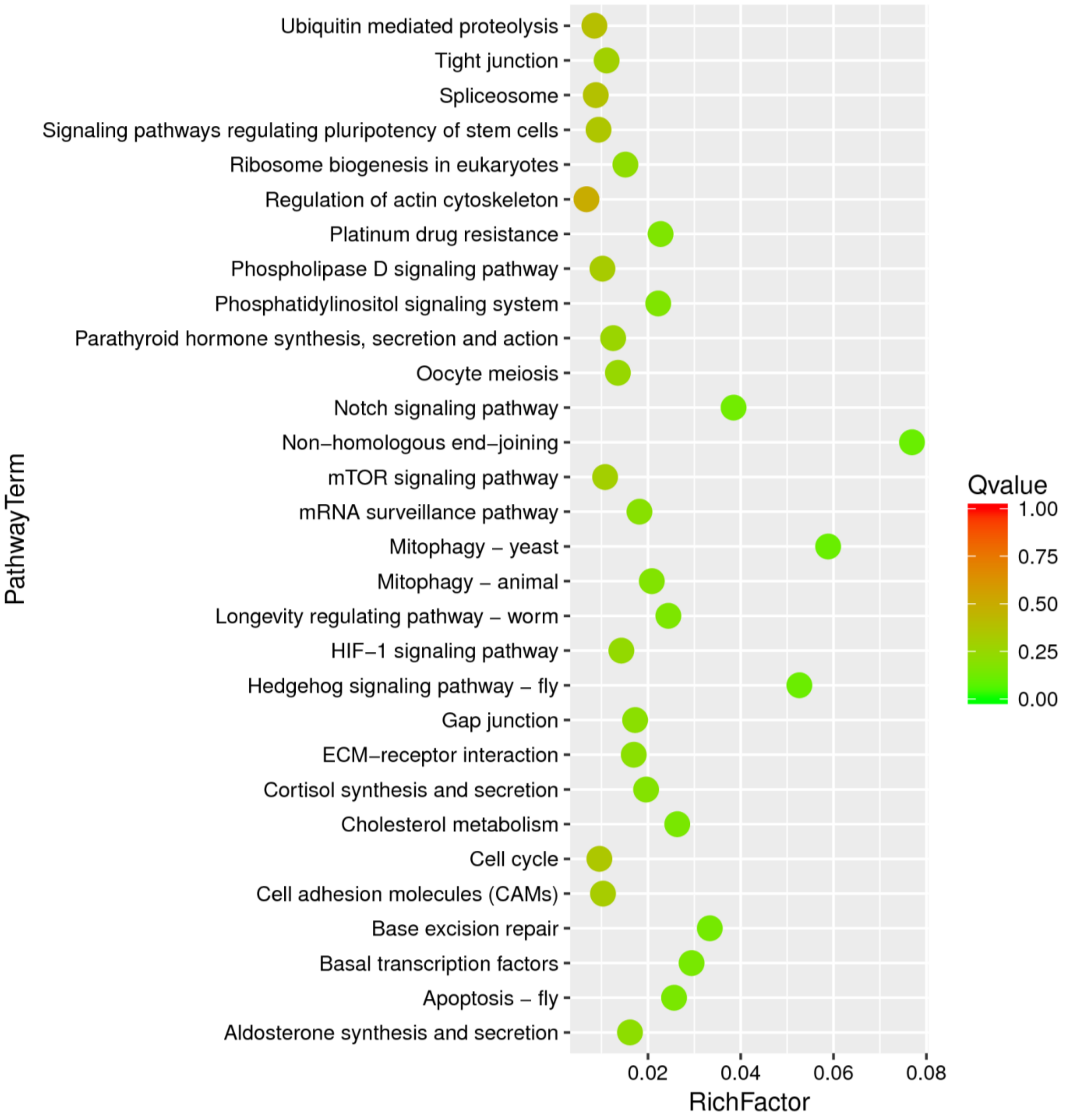

Supplemental Figure 6. Pur-α may regulate multiple genes of diverse pathways in T-*Dusp8* cKO T cells. Kyoto Encyclopedia of Genes and Genomes (KEGG) enrichment analysis shows the pathways of multiple peak-associated genes identified by Pur-α-ChIP sequencing using T-*Dusp8* cKO T cells compared to those of wild-type (*DUSP8*$^{f/f}$) T cells.

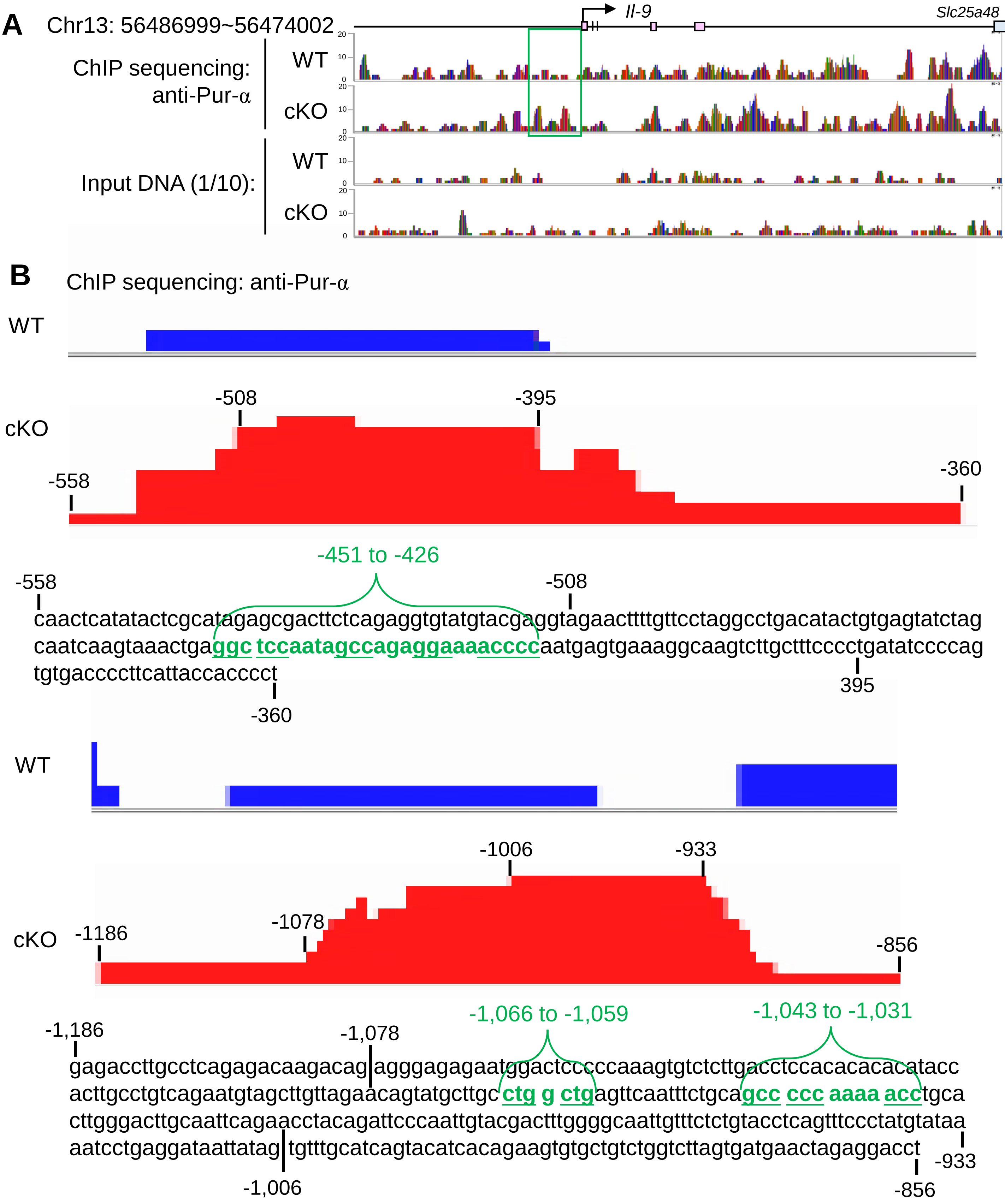

Supplemental Figure 7. Sequences of enhanced Pur-α-binding regions in the mouse *Il-9* promoter. **(A)** The binding of Pur-α to the IL-9 gene locus in TGF-β-stimulated T cells from T-*Dusp8* cKO or wild-type mice was analyzed by chromatin-immunoprecipitation (ChIP) sequencing using anti-Pur-α antibody. The peaks showed the Pur-α-binding regions (GRCm38.p6 murine reference genome Chr13: 56486999~56474002). **(B)** Characters shown in green denote the three putative Pur-α-binding elements (-451 to -426, -1,043 to -1,031, and -1,066 to -1,059) coinciding with the two regions that showed the most enhanced Pur-α bindings to the *Il-9* promoter in T-*Dusp8* cKO T cells. Characters with a underline denote the putative Pur-α-binding repeats, (GGN)n or (CAG)n motif, as well as its complementary sequence (NCC)n or (CTG)n. N denotes any nucleotide.

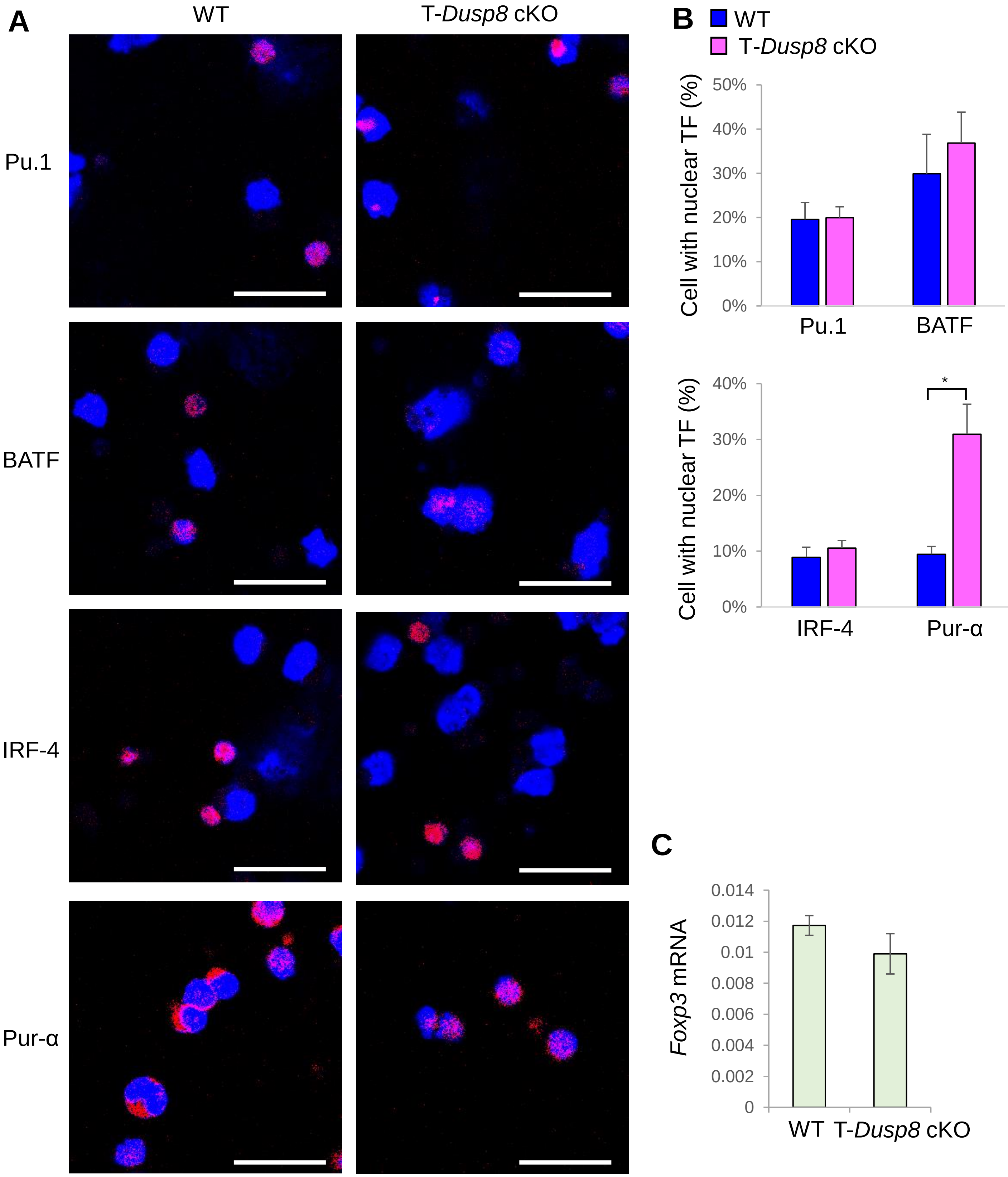

Supplemental Figure 8. DUSP8 regulates Th9 differentiation in a PU.1/BATF/IRF-4/Foxp3-independent manner. (**A**) Confocal microscopy analyses of PU.1, BATF, IRF-4, or Pur-α in the in vitro differentiated Th9 cells of wild-type or T-*Dusp8* cKO mice. Cell nuclei were stained with DAPI. Scale bars, 25 μm. (**B**) The percentages of T cells containing the nuclear PU.1, BATF, IRF-4, or Pur-α were shown as mean ± SEM from 5 images. TF, transcription factor. (**C**) Real-time PCR analyses of *Foxp3* mRNA levels in the *in vitro* differentiated Th9 cells of wild-type or T-*Dusp8* cKO mice. *Foxp3* mRNA levels were normalized to *Srp72* levels. n = 2 (technical replicates). Means ± SD are shown. *, *P* value < 0.05 (two-tailed Student's t-test)

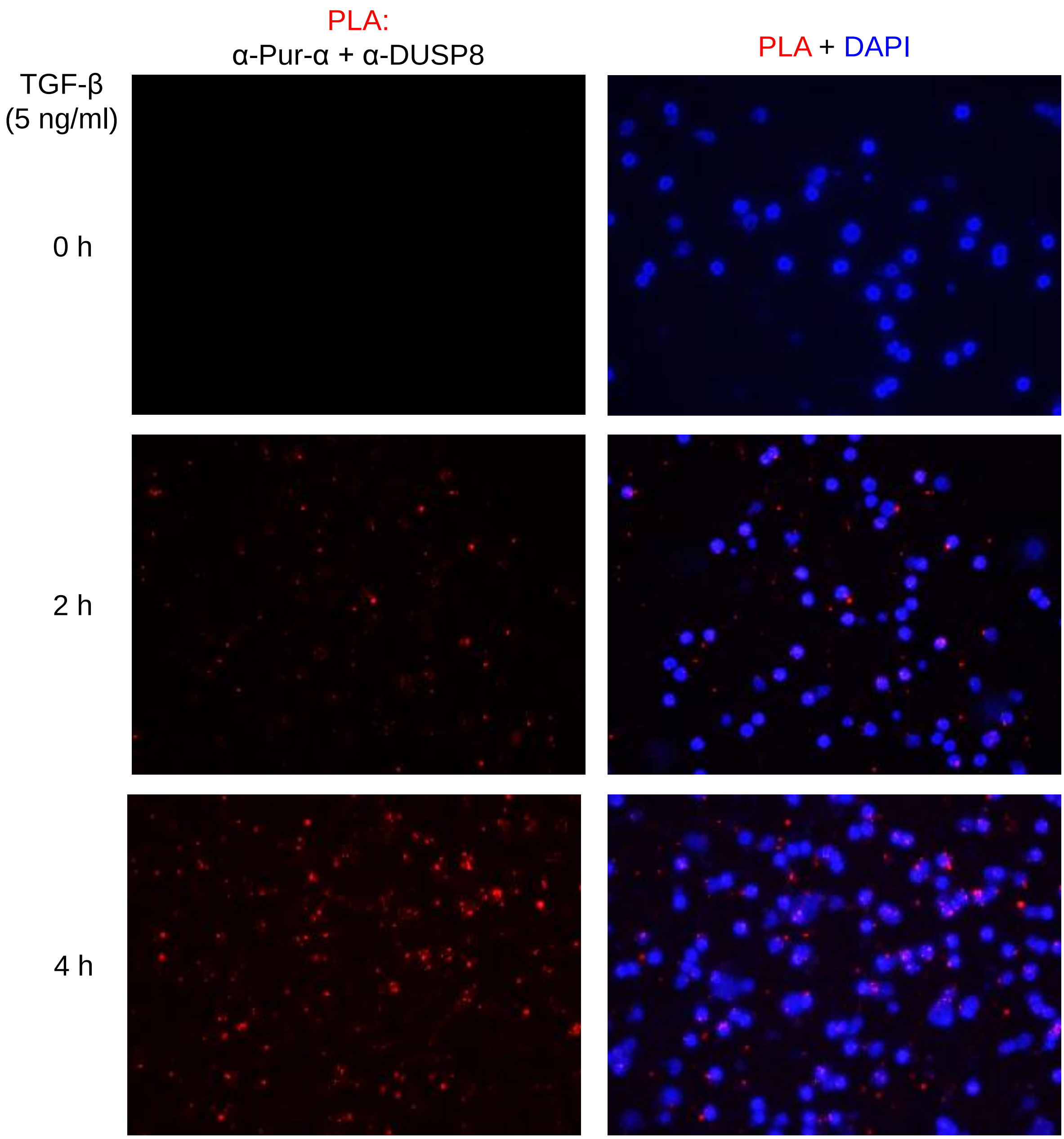

Supplemental Figure 9. Pur-α directly interacts with DUSP8 in T cells stimulated with TGF-β. In situ proximity ligation assays (PLA) of close proximity (< 40 nm) between Pur-α and DUSP8 in murine primary T cells stimulated with TGF-β (5 ng/ml) for 6 h. For PLA, red dots represent direct interaction signals. Cell nuclei were stained with DAPI. Original magnification, x630.

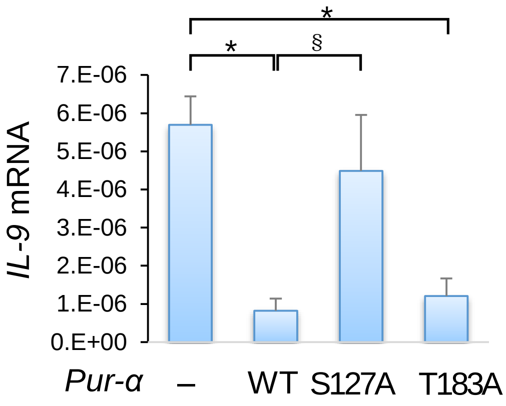

Supplemental Figure 10. Pur-α overexpression inhibits *IL-9* mRNA levels in Jurkat T cells stimulated with TGF-β. Real-time PCR of *IL-9* mRNA levels in Jurkat T cells transfected with *Pur-α* wild-type or phospho-deficient mutant (S127A or T183A) plasmid. Jurkat cells were stimulated with TGF-β (50 ng/ml) for 8 h. The expression levels of *IL-9* mRNAs were normalized to *GAPDH* levels. *, *P* value < 0.05 (one-way ANOVA and Tukey's post hoc test); §, *P* value < 0.05 (one-way ANOVA and least significance difference test).

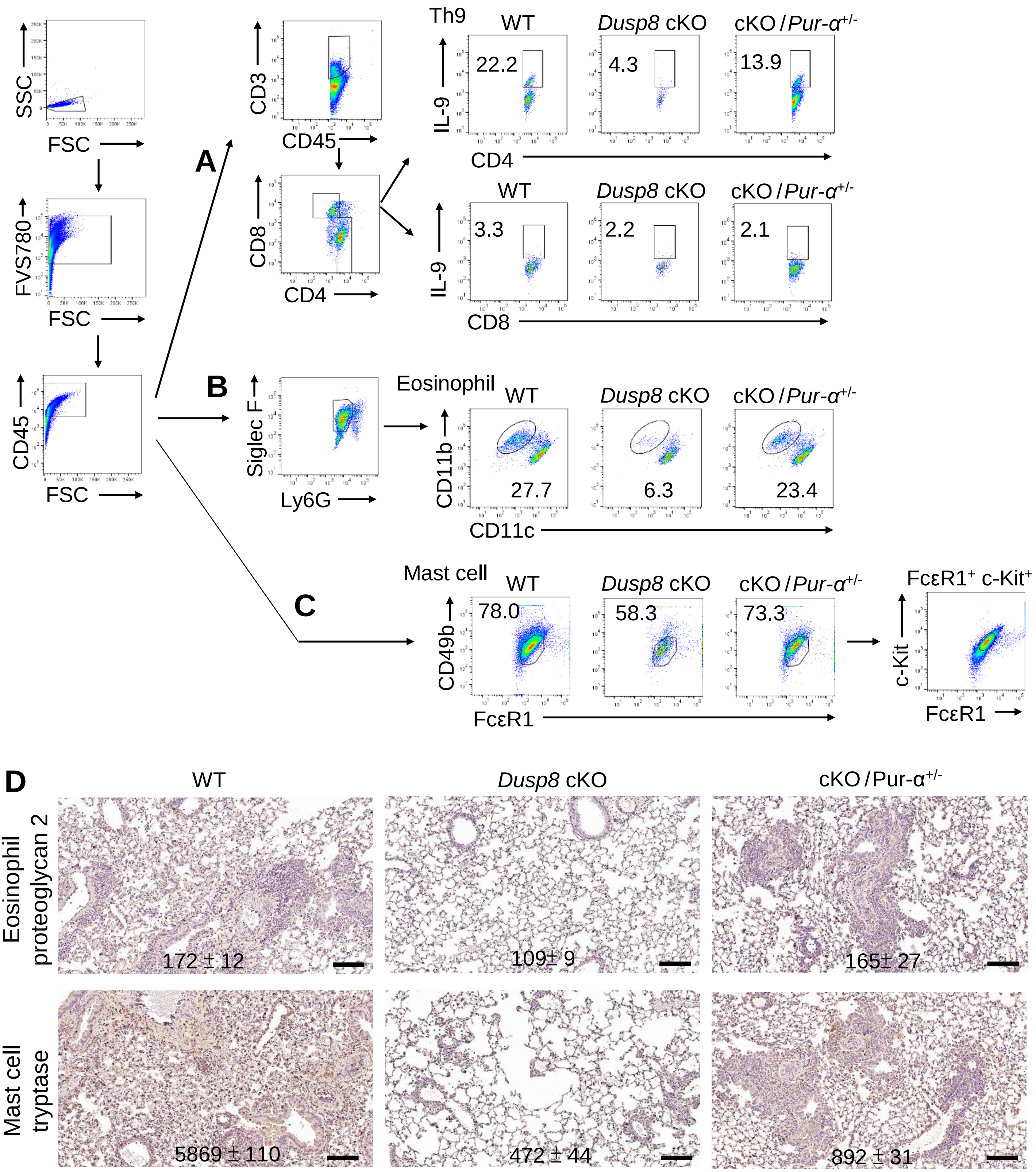

Supplemental Figure 11. Lung infiltrating immune cells of OVA-induced allergic asthma are decreased in T-cell-specific *Dusp8* conditional knockout mice. (**A** to **C**) Flow cytometry analyses of Th9 cells (CD45$^+$, CD3$^+$, CD4$^+$, IL-9$^+$), IL-9-producing CD8$^+$ T cells (CD45$^+$, CD3$^+$, CD8$^+$, IL-9$^+$) (A), eosinophils (CD45$^+$, Ly6G$^-$, CD11b$^+$, CD11c$^-$) (B), and mast cells (CD45$^+$, CD49b$^-$; the infiltrating mast cells are also c-kit$^+$, FcεR1$^+$) (C) in the lungs from mice on day 26 of the OVA-induced allergic asthma. Infiltrating immune cells were isolated from the lung tissues by Percoll gradient centrifugation. (**D**) Immunohistochemical staining images of mast cell tryptase (a mast-cell marker) and proteoglycan 2 (also named pro-eosinophil major basic protein, an eosinophil marker) in the lung tissues of OVA-induced allergic asthma mice. Mean ± SEM of infiltrating immune cells from 3 images are shown below individual panels. Scale bars, 100 μm. Data shown are a representative of *Dusp8*$^{f/f}$ (WT), *Dusp8*$^{f/f}$;*Cd4-Cre* (*Dusp8* cKO), and *Dusp8*$^{f/f}$;*Cd4-Cre*;*Pur-α*$^{+/-}$ (cKO / *Pur-α*$^{+/-}$).

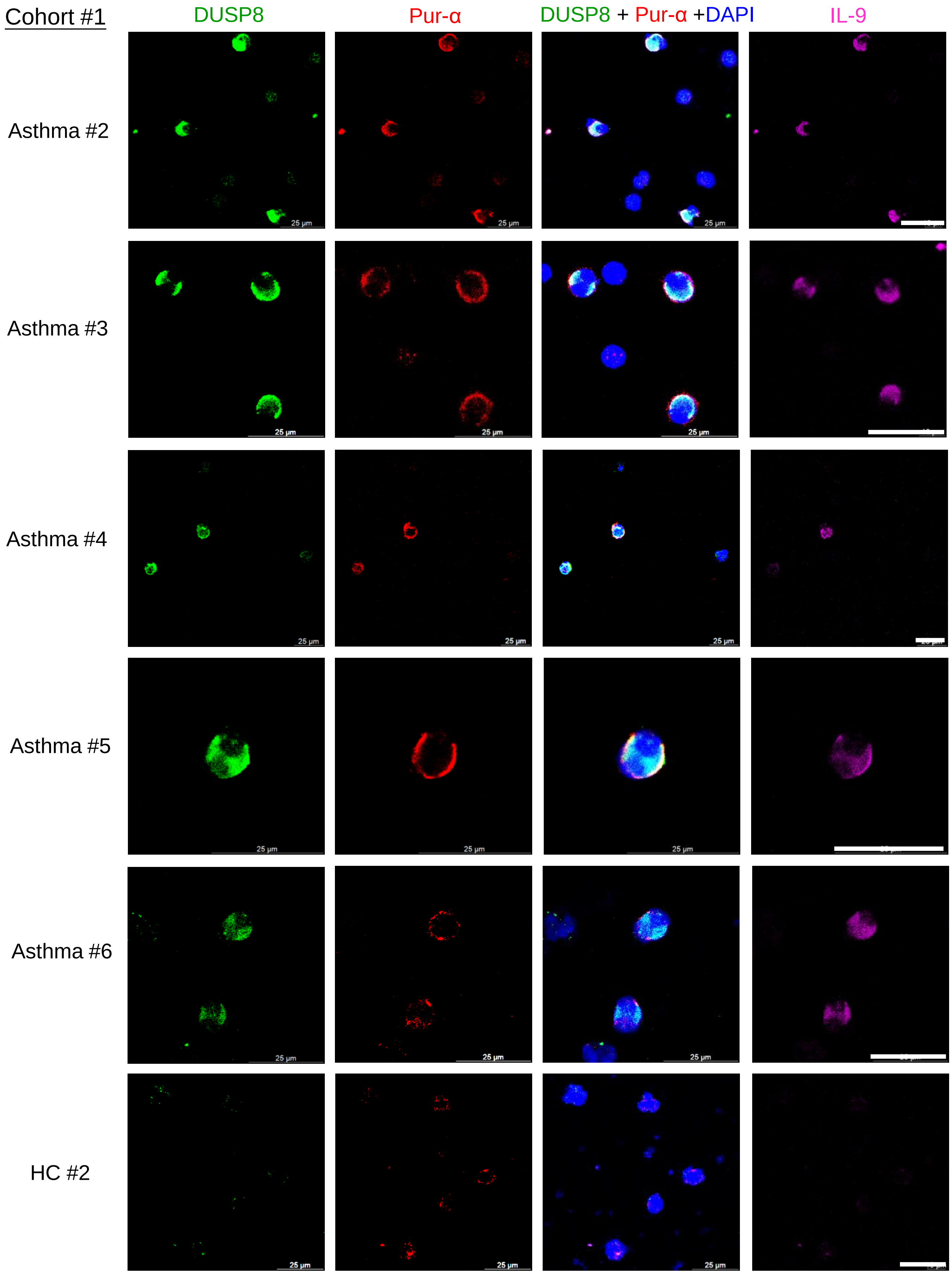

Supplemental Figure 12. Pur-α mainly localizes to the cytoplasm of the T cells from patients with asthma or atopic dermatitis. Confocal microscopy analyses of DUSP8 (green), Pur-α (red), DAPI (blue), and IL-9 (purple) in the peripheral blood T cells of 11 representative healthy controls (HC), 22 representative asthma patients, and 7 representative atopic dermatitis (AD) patients from Cohort #1 and Cohort #2. Scale bars, 25 µm.

(Continued Supplemental Figure 12)

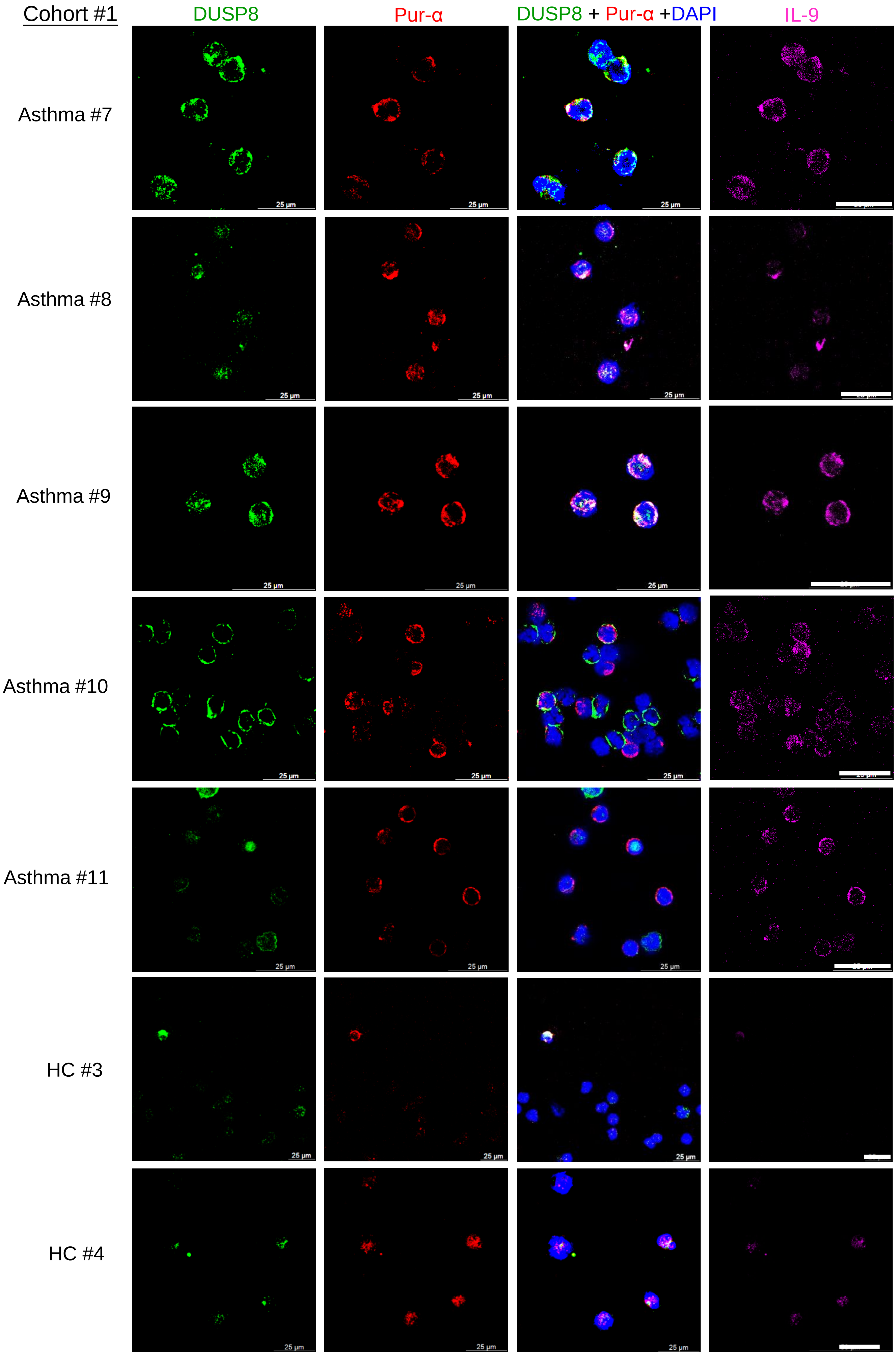

(Continued Supplemental Figure 12)

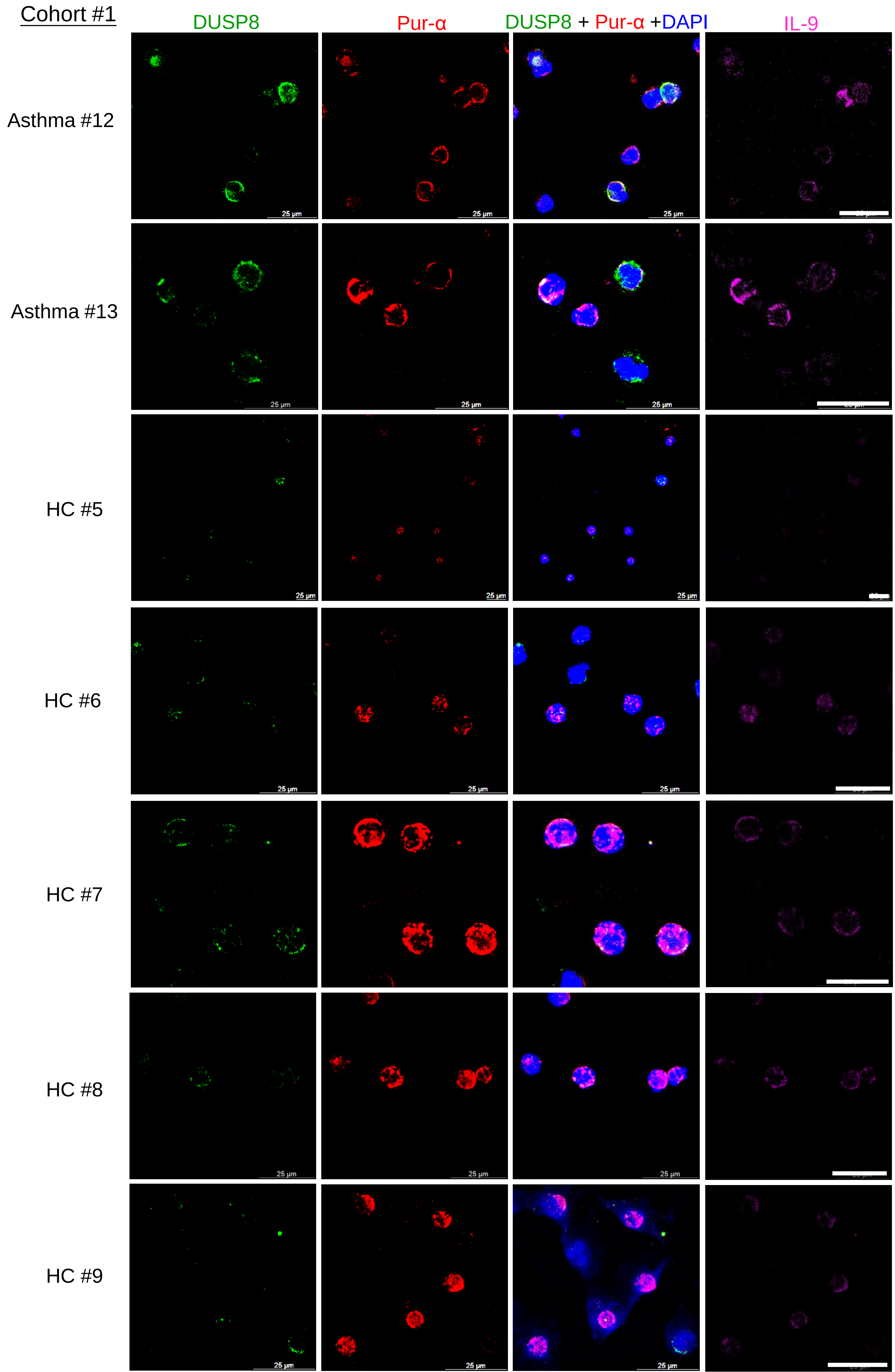

(Continued Supplemental Figure 12)

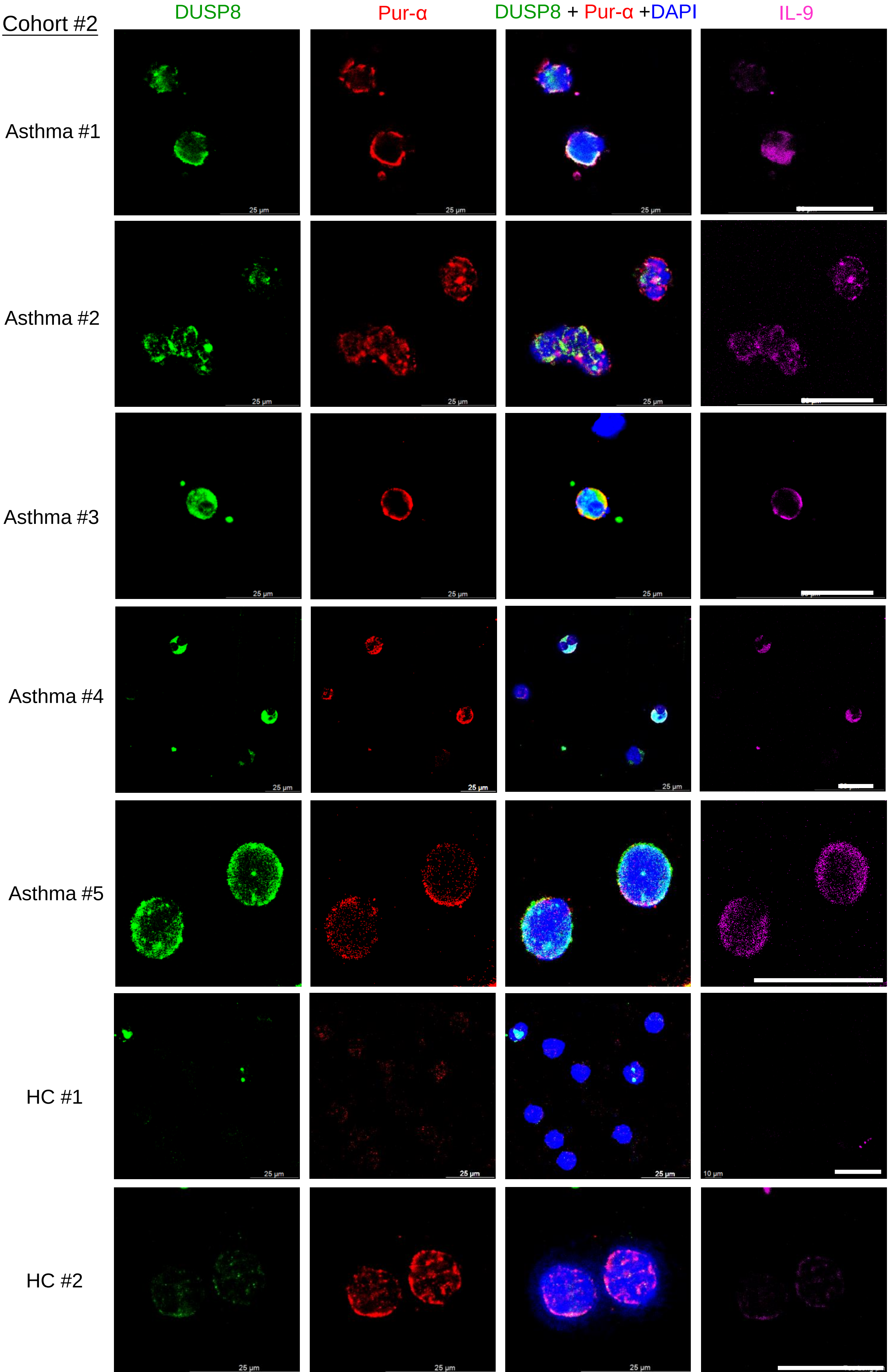

(Continued Supplemental Figure 12)

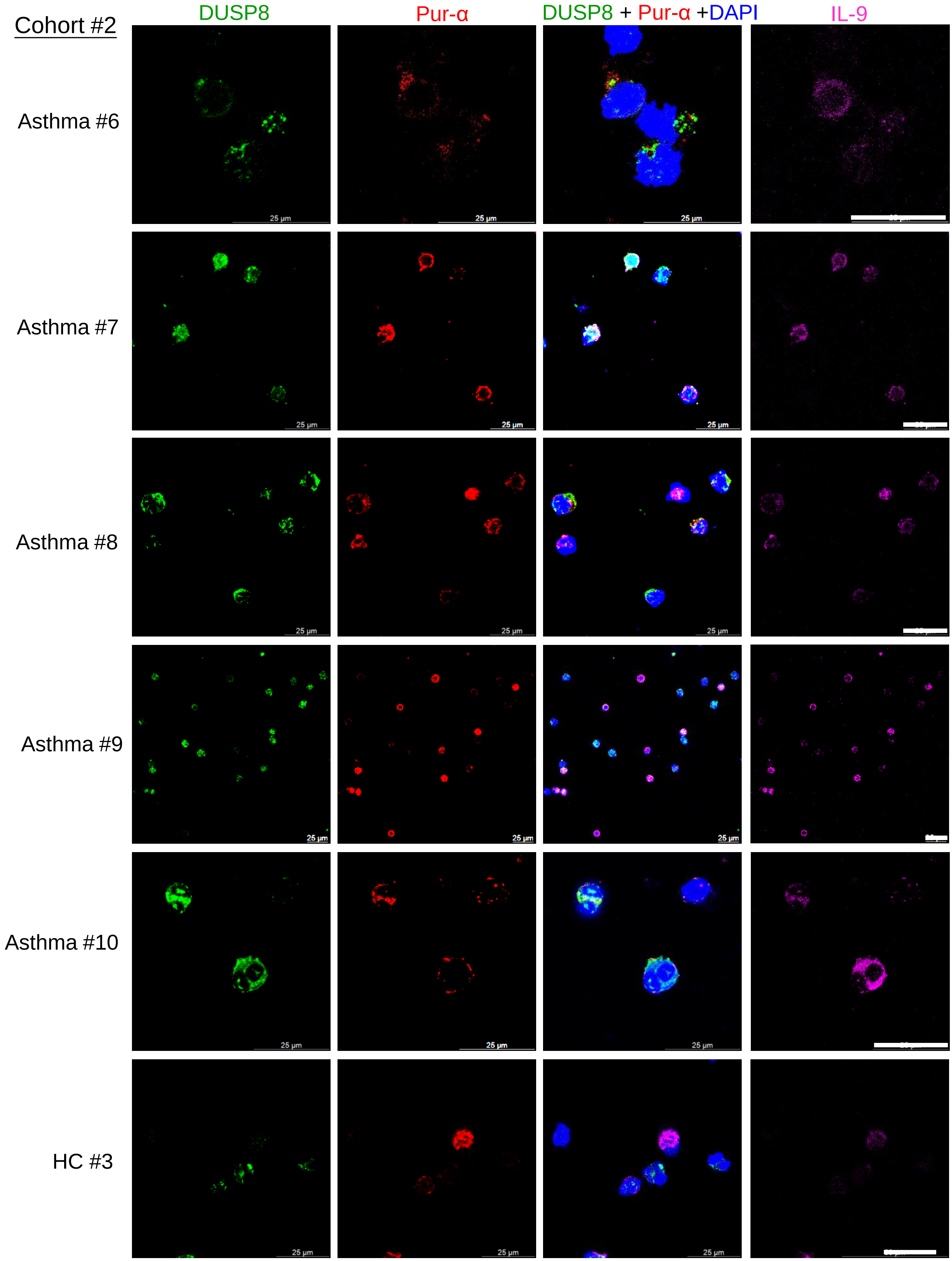

(Continued Supplemental Figure 12)

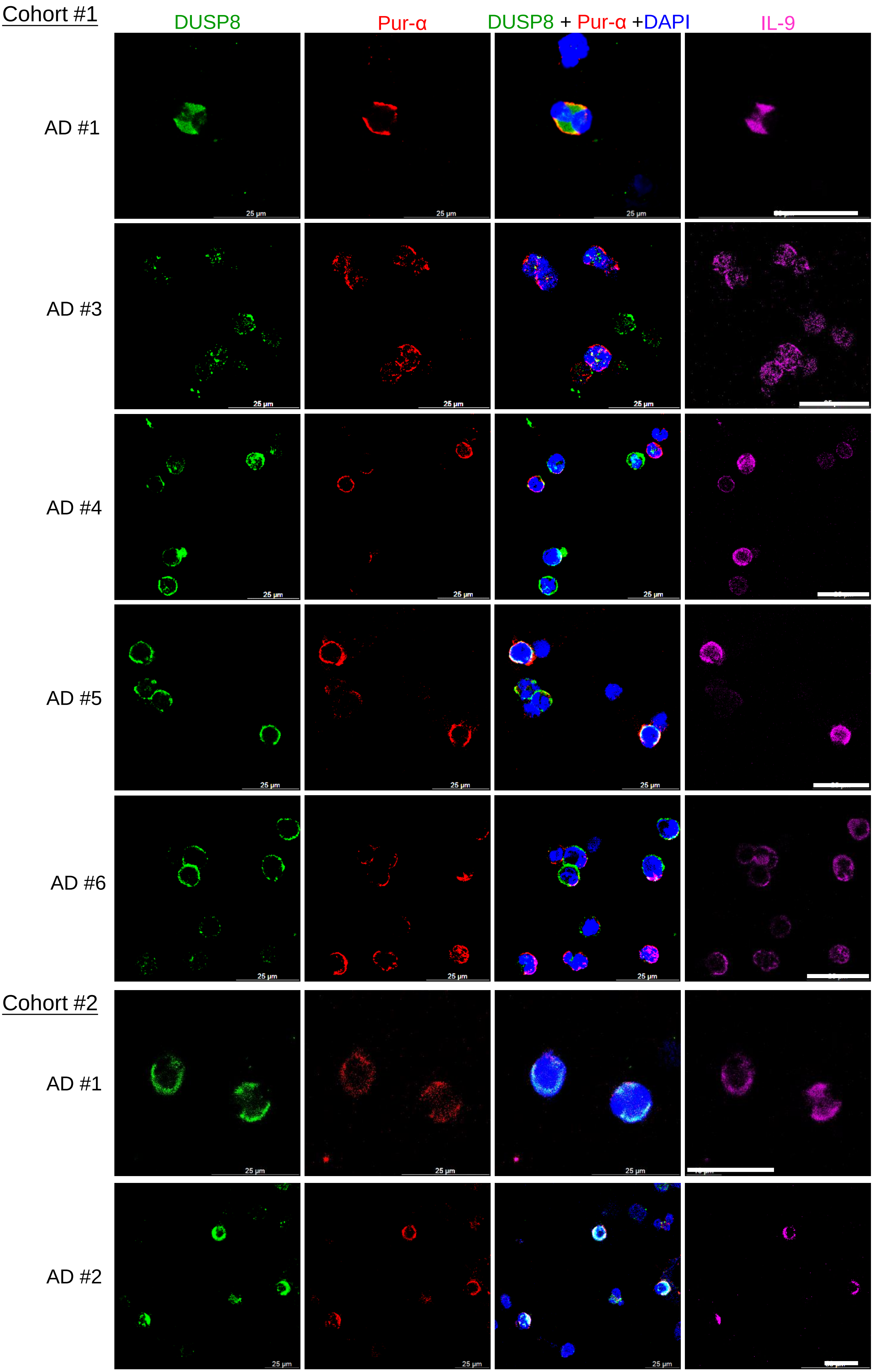

Supplemental Table 1. Cohort #1 Profile of asthma patients, atopic dermatitis patients, and healthy controls

**Cohort #1**	**Asthma (total n = 16)**		**HC (total n = 18)**		**AD (total n = 6)**	
**Clinical data**	**n or Median**	**% or (Q1-Q3)**	**n or Median**	**% or (Q1-Q3)**	**n or Mean**	**% or (SEM)**
Female, n	n = 10	62.5%	n = 18	100%	n = 2	33.3%
Age (year-old)	41	(34.5-47.5)	32.0	(24.0-39.0)	20.8	3.26
ACT	23.5	(23-24)	N/A		N/A	
Atopic dermatitis	n = 4	25.0%	N/A		N/A	
Allergic rhinitis	n = 7	43.8%	N/A		N/A	
Allergic conjunctivitis	n = 1	6.3%	N/A		N/A	
IgE (IU/ml)	120.6	(39.3-324.8)	N/A		3517.0	1382.5
EASI	N/A		N/A		18.34	8.09

HC, healthy controls; AD, atopic dermatitis; Q1, the first quartile; Q3, the third quartile; SEM, standard error of the mean; N/A, not applicable; ACT, asthma control test; EASI, eczema area and severity index.

Supplemental Table 2. Cohort #2 Profile of asthma patients, atopic dermatitis patients, and healthy controls

Cohort #2	Asthma (total n = 11)		HC (total n = 5)		AD (total n = 2)	
**Clinical data**	**n or Median**	**% or (Q1-Q3)**	**n or Median**	**% or (Q1-Q3)**	**n or Mean**	**% or (SEM)**
Female, n	n = 8	72.7%	n = 3	60.0%	0	0%
Age (year-old)	46	(38.0-50.5)	37.0	(31.0-39.0)	27.5	15.5
ACT	21.0	(20.0-22.5)	N/A		N/A	
IgE (IU/ml)	171.0	(36.8-200.0)	N/A		2813	2187
EASI	N/A		N/A		6.05	3.15

HC, healthy controls; AD, atopic dermatitis; Q1, the first quartile; Q3, the third quartile; SEM, standard error of the mean; N/A, not applicable; ACT, asthma control test; EASI, eczema area and severity index.
